# Inflammatory cytokine IL6 cooperates with CUDR to aggravate hepatocyte-like stem cells malignant transformation through NF-κB signaling

**DOI:** 10.1038/srep36843

**Published:** 2016-11-11

**Authors:** Qidi Zheng, Zhuojia Lin, Xiaonan Li, Xiaoru Xin, Mengying Wu, Jiahui An, Xin Gui, Tianming Li, Hu Pu, Haiyan Li, Dongdong Lu

**Affiliations:** 1 School of Life Science and Technology, Tongji University, Shanghai, 200092, China

## Abstract

Inflammatory cytokines and lncRNAs are closely associated with tumorigenesis. Herein, we reveal inflammatory cytokines IL6 cooperates with long noncoding RNA CUDR to trigger the malignant transformation of human embryonic stem cells-derived hepatocyte-like stem cells. Mechanistically, IL6 cooperates with CUDR to cause MELLT3 to interact with SUV39h1 mRNA3′UTR and promote SUV39h1 expression. Moreover, the excessive SUV39h1 also increases tri-methylation of histone H3 on nineth lysine (H3K9me3). Intriguingly, under inflammatory conditions, H3K9me3 promotes the excessive expression and phosphorylation of NF-κB, and in turn, phorsphorylated NF-κB promotes the expression and phosphorylation of Stat3. Furthermore, that the phosphorylated Stat3 loads onto the promoter region of miRs and lncRNAs. Ultimately, the abnormal expression of miRs and lncRNAs increased telomerase activity, telomere length and microsatellite instability (MSI), leading to malignant transformation of hepatocyte-like stem cells.

Human embryonic stem cell lines were differentiated into endoderm-like cells and further differentiated to hepatocyte-like stem cells^1^,^2^,^3^,^4^. Because the tumor microenvironment plays a critical role in cancer progression, chronic inflammation is an important process leading to tumorigenesis. Inflammation is often caused by a variety of inflammatory cytokine, e.g. the interleukin (IL)-6, a pleiotrophic cytokine known to be involved in the tumorigenesis^5^. A report indicated Predominant activation of JAK/STAT3 pathway by Interleukin-6 was implicated in hepatocarcinogenesis^6^.

Long non-coding RNAs (lncRNAs) have been shown to have important regulatory roles in cancer biology^7^,^8^. Cancer up-regulated drug resistant (Urothelial cancer associated 1, UCA1, CUDR) is a novel non-coding RNA gene which is upregulated in several cancers, such as bladder cancer, breast cancer and colorectal cancer, and plays a pivotal role in cancer progression^9^,^10^. In particular, CUDR activates mTOR to regulate hexokinase 2 HK2 through both activation of STAT3 and repression of mir-143^11^,^12^. Moreover, CUDR regulates mRNA stability, drug resistance, apoptosis process and Akt signaling pathway^13^,^14^,^15^. Moreover, CUDR regulated cell cycle through CREB via PI3K-AKT dependent pathway in bladder cancer^16^, and had important implications in postoperative noninvasive follow-up^17^ and during human liver stem cell malignant differentiation^18^,^19^. N(6)-methyladenosine (m^6^A) is the most prevalent internal (non-cap) modification present in the messenger RNA of all higher eukaryotes and regulates mRNA stability^20^. Furthermore, the m(6)A mark acts as a key post-transcriptional modification that promotes the initiation of miRNA biogenesis^21^. In particular, m6A mRNA methylation facilitates resolution of naïve pluripotency toward differentiation^22^. NF-κB is a key transcriptional regulator involved in inflammation and cell proliferation, survival, and transformation^23^,^24^. For example, Metformin suppresses pancreatic tumor growth with inhibition of NFκB/STAT3 inflammatory signaling^25^. Moreover, YM155 potently triggers cell death in breast cancer cells through an autophagy-NF-kB network^26^. In addition, a report indicated that STAT3 could promote epithelial to mesenchymal transition in breast cancer cells^27^.

In the present study, we demonstrate that interplay between CUDR with inflammatory cytokines prompts the malignant transformation of hepatocyte-like cells induced by human embryonic stem cells. These observations provide insight into a novel link between CUDR and inflammatory cytokines in hepatocarcinogenesis.

## Results

### CUDR prompts malignant transformation of human embryonic stem cells derived-hepatocyte-like stem cells in mouse injury liver inflammation microenvironment

**Figure 1:**
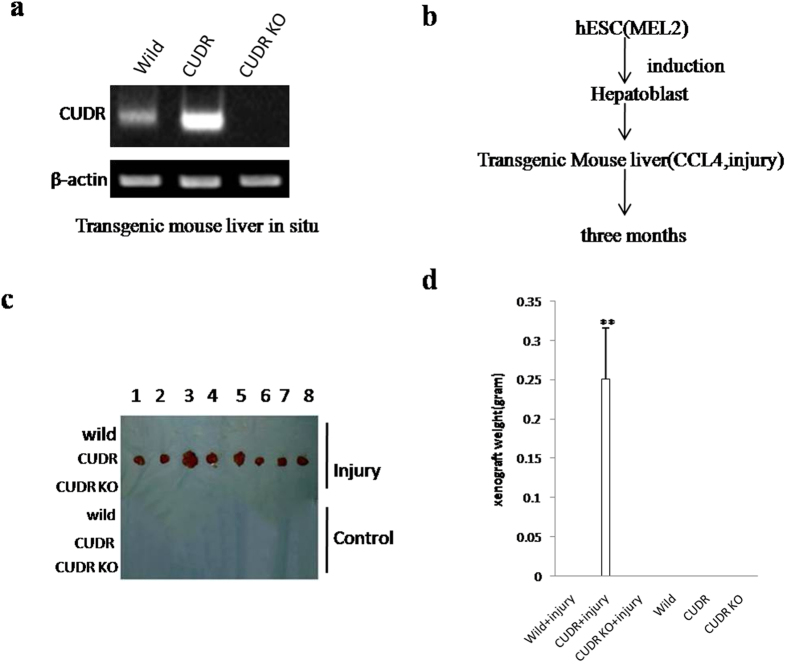
The hepatoblast derived from human ES cells were subjected to transform onto liver cancer in CUDR overexpressed and CCL4 treated injury mouse liver. (**a**) Mouse liver *in vivo* transfection of CUDR overexpression or knockdown plasmids. The RT-PCR analysis with CUDR primers in these mice liver tissue. β-actin as internal control. (**b**) The schematic diagram illustrates a model of human stem cell line MEL-2 differentiation into hepatoblast. Then the hepatoblasts were injected into the liver capsule under the B ultrasound guide. CUDR overexpressed/knocked-down and CCL4 induce injury mice Liver *In Vivo* Transfection. (**c**) The mice were stratified and the tumors were recovered. (**d**) The wet weight of each tumor was determined for each mouse. Each value was presented as mean ± standard error of the mean (SEM). **P < 0.01 (**b**).

To explore whether excessive CUDR prompts malignant transformation of human embryonic stem cells derived-hepatocyte-like stem cells in the injury liver inflammation microenvironment induced with Carbon tetrachloride (CCl4), induced hepatotoxicity and trigger liver injury^28^,^29^, we performed liver multiple transfection *in vivo* with CUDR overexpression or knockdown plasmids. Compared with wild-type mice, pCMV6-A-GFP-CUDR multiple transfected mouse expressed excessive CUDR, as well as CUDR was knocked down in pGFP-V-RS-CUDR multiple transfected mouse (Fig. 1a). Next, we selected human embryonic stem cells derived- hepatoblasts (HESCDHs) for experiments according to the schematic diagram illustration (Fig. 1b). These HESCDHs were inoculated into the Mouse liver capsule under the B ultrasound guide, including six groups: wild, CUDR, CUDR KO, wild plus CCL4, CUDR plus CCL4, CUDR KO plus CCL4. The CCL4 treatment plus CUDR excessive HESCDHs were transformed into tumor in the mouse injury liver (0.251 ± 0.065 gram, n = 8), as well as the tumor was not produced in the rest groups at all (Fig. 1c,d). Furthermore, histological hematoxylin-eosin (HE) staining identified these transformed tumor were malignant liver cancer (Figure S1A). In addition, the AFP (a liver cancer marker) was expressed in the liver tumor tissue (Figure S1B). Taken together, these observations suggest that excessive CUDR can trigger HESCDHs malignant transformation in mouse injury liver inflammatory environment.

### CUDR accelerates hepatocyte-like stem cells malignant transformation in coordination with inflammatory cytokine IL6

**Figure 2:**
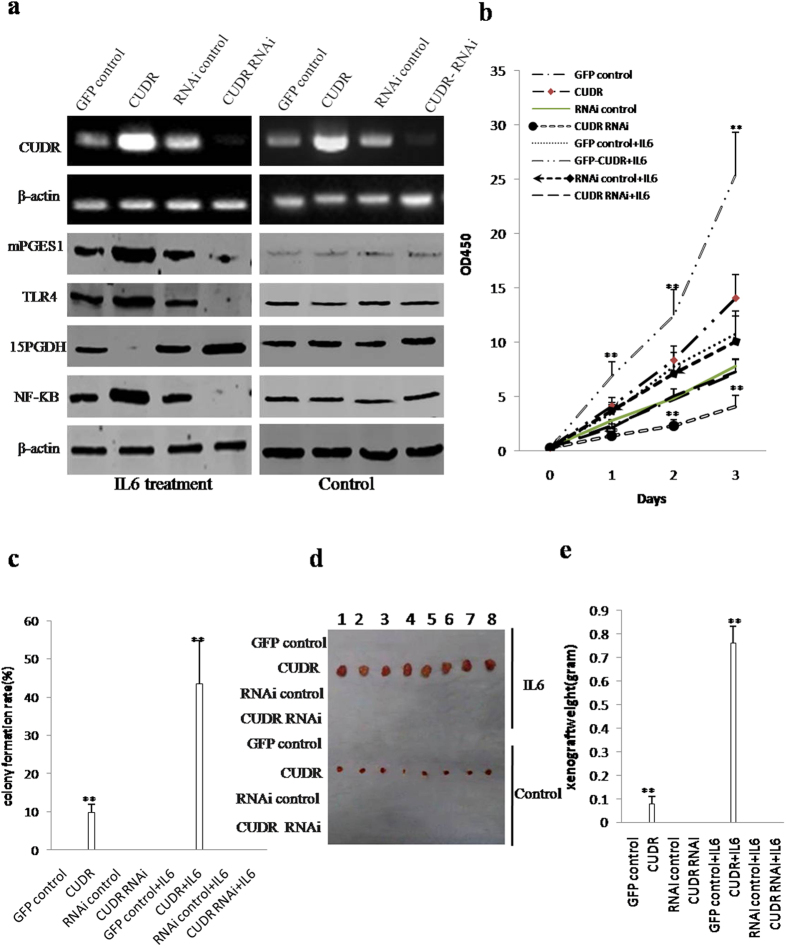
CUDR overexpression combined with inflammatory factor IL6 produced hepatocyte-like stem cells malignant transformation *in vitro* and *in vivo*. (**a**) RT-PCR analysis for CUDR and Western blotting analysis with anti-mPGES1, anti-TLR4, anti-15PGDH and anti-NF-κB in the hepatocytes-like stem cells. β-actin as internal control. (**b**) *In vitro* test in induced and treated hepatocytes–like stem cells. Cells growth assay using CCK8. Each value was presented as mean ± standard error of the mean (SEM). **P < 0.01. (**c**) Cells soft agar colony formation assay. (**d**) *In vivo* test in induced and treated hepatocytes–like stem cells. The mice were stratified and the tumors were recovered. The photography of xerograft tumor in the four groups (indicated in *left)*. (**e**) The wet weight of each tumor was determined for each mouse. Each value was presented as mean ± standard error of the mean (SEM), **P < 0.01.

To validate whether the role of CUDR in malignant transformation of hepatocyte-like stem cells needs the help of inflammatory cytokines IL6, we designed the experimental lines (Figure S2A). We first established MEL-2 cell lines with stable overexpression or depletion of CUDR. and induced these transfected MEL-2 cells into hepatoblasts. As shown Fig. 2a, the level of CUDR was increased in pCMV6-A-GFP-CUDR+IL6 (10 μM) and pCMV6-A-GFP-CUDR group and reduced in pGFP-V-RS-CUDR+IL6 (10 μM) and pGFP-V-RS-CUDR group compared to control. In IL6 treated groups, excessive CUDR enhanced and CUDR knockdown reduced the mPGES1, TLR4, NF-κB expression, as well as CUDR overexpression reduced and CUDR knockdown increased the 15PGDH expression in these induced hepatocyte-like stem cells. On the other hand, mPGES1, TLR4, NF-κB, 15PGDH expression were significantly not changed in IL6 untreated groups.

Next, we examined the growth curves of the eight hepatocyte-like stem cell lines. As shown in Fig. 2b, in derived hepatocyte-like stem cells treated with IL6, CUDR overexpression significantly increased and CUDR knockdown significantly inhibited the growth of hepatocyte-like stem cells when compared to the control cells. In derived hepatocyte-like stem cells untreated with IL6, CUDR overexpression also significantly increased and CUDR knockdown significantly inhibited the growth of hepatocyte-like stem cells when compared to the control cells. However, this potential role of excessive CUDR was increased in IL6 treated group than in IL6 untreated group. To further address this issue, we detected the S phage cells by BrdU staining. As shown in Figure S2A, after these induced hepatocyte-like stem cells were treated with IL6, excessive CUDR significantly increased (89.3 ± 21.3% vs 36.7 ± 8.4% p < 0.01) and CUDR knockdown significantly inhibited (10.3 ± 2.1% versus 31.6 ± 5.2%, p < 0.01) the BrdU positive rate when compared to the control cells. Although either CUDR overexpression (54.6 ± 11.2% versus 23.4 ± 4.2%, P < 0.05) or knockdown (10.3 ± 2.1% versus 20.1 ± 3.5%, P < 0.05) did significantly alter the BrdU positive rate when these derived-hepatocyte-like stem cells were not treated with IL6, the altered capacity was significantly attenuated compared to the IL6 treated hepatocyte-like stem cells. We further performed soft-agar colony formation assay, as shown in Fig. 2c, The soft-agar colony-formation rate was 43.5 ± 11.2% in the pCMV6-A-GFP-CUDR+IL6 group (P < 0.01) and 9.7 ± 2.2% in the pCMV6-A-GFP-CUDR group (P < 0.01), however, the soft-agar colony was not exhibited in the rest groups (P < 0.01). Furthermore, the hepatocyte-like stem cells were plated in stem cell conditioned culture medium which allowed for the formation of colonies separated from each other. As shown in Figure S2C, after these derived-hepatocyte-like stem cells were treated with IL6, CUDR overexpression significantly increased (68.2 ± 15.3% vs 12.3 ± 2.7% p < 0.01) and CUDR knockdown significantly inhibited (5.4 ± 1.2% versus 11.9 ± 2.4%, p < 0.01) the self-renewing sphere-formation rate when compared to the control cells. Although either CUDR overexpression (20.5 ± 4.2% versus 10.2 ± 2.1%, P < 0.05) or knockdown (4.1 ± 0.82% versus 8.9 ± 1.8%, P < 0.05) did significantly alter the number of self-renewing sphere-formation rate when these derived hepatocyte-like cells were not treated with IL6, the capacity was significantly attenuated compared to that of the IL6 treated hepatocyte-like cells. To further determine whether CUDR plus IL6 promotes hepatocyte-like cells malignant transformation *in vivo* cooperatively, the aforementioned eight group of cell lines were injected subcutaneously at armpit into athymic Balb/C mice. The findings showed that the excessive CUDR and IL6 treated simultaneously hepatocyte-like stem cells were transformed into xenograft tumors (0.76 ± 0.074 gram, n = 8, P < 0.01) and the excessive CUDR hepatocyte-like stem cells also were transformed into xenograft tumors (0.0775 ± 0.032 gram, n = 8, P < 0.01), as well as the xenograft tumor was not appeared in the rest groups at all (Fig. 2d,e). Furthermore, pathological examination (H&E staining) of the xenograft tumors revealed a poorly-differentiated tumor cells (Figure S2D). Taken together, CUDR combined with IL6 collaboratively aggravates derived-hepatocyte- like stem cells malignant transformation and proliferation.

### CUDR enhances the expression of suppressor of variegation 3–9 homolog 1 (**SUV39H1**), a histone methyltransferase, dependent on IL6

**Figure 3:**
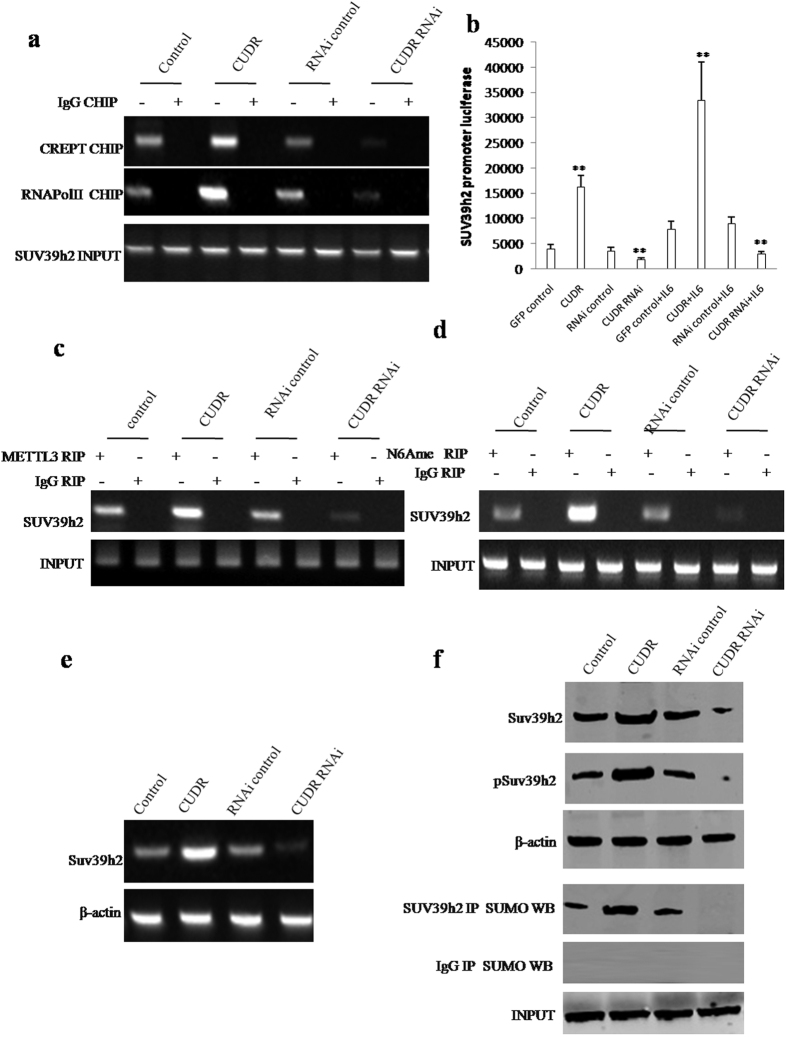
CUDR enhances SUV39h2 expression in hepatocyte-like stem cells with IL6 treatment. (**a**) Chromatin Immunoprecipitation (CHIP) with anti-CREPT, anti-RNA PolII followed by PCR with SUV39h2 promoter primers in hepatocyte-like stem cells treated with IL6 and transfected with pCMV6-A-GFP, pCMV6-A-GFP-CUDR, pGFP-V-RS, pGFP-V-RS-CUDR. IgG CHIP as negative control. SUV39h2 promoter DNA as INPUT. (**b**) SUV39h2 promoter luciferase activity assay. Each value was presented as mean ± standard error of the mean (SEM). **P < 0.01. (**c**) RNA Immunoprecipitation (RIP) with anti-METTL3 followed by RT-PCR with SUV39h2 primers. IgG RIP as negative control. SUV39h1 mRNA as INPUT. (**d**) RNA Immunoprecipitation (RIP) with anti-N6Ame followed by RT-PCR with SUV39h2 mRNA primers. IgG RIP as negative control. SUV39h1 mRNA as INPUT. (**e**) SUV39h2 expression analysis by RT-PCR. β-actin as internal control. (**f**) The analysis of SUV39h2 expression, SUV39h2 phosorylation and SUV39h2 sumoylation by western blotting with anti-SUV39h2, anti-pSUV39h2, β-actin as internal control, or IP with anti-SUV39h2 and anti-SUM western blotting. Western blotting with anti-SUV39h2 was uesd as INPUT.

To elucidate whether CUDR may enhance SUV39h2 expression via IL6, we first performed Chromatin Immunoprecipitation (CHIP) to detect the transcriptional activity of SUV39h2. As shown in Fig. 3a, CUDR overexpression facilitated and CUDR knockdown reduced the loading of CREPT ((***c***ell-cycle ***r***elated and ***e***xpression- elevated ***p***rotein in ***t***umor) and RNA polymerase II onto the SUV39h2 promoter region. Moreover, CUDR overexpression increased and CUDR knockdown decreased the SUV39h2 promoter luciferase activity (Fig. 3b). Furthermore, CUDR overexpression enhanced and CUDR knockdown inhibited the METTL3 expression. However, CUDR overexpression or knockdown did not alter the FTO expression in IL6 treated hepatocyte-like cells (Figure S3A). Intriguingly, RNA Immunoprecipitation (RIP) with anti-METTL3 showed that CUDR overexpression enhanced and CUDR knockdown inhibited the binding of METTL3 to SUV39h2 mRNA (Fig. 3c). Furthermore, RNA Immunoprecipitation (RIP) with anti-N6Ame showed that CUDR overexpression enhanced and CUDR knockdown inhibited the mRNA methylation of SUV39h2 (Fig. 3d). Super-EMSA (gel-shift) (Figure S3B) and Nuclear Run-on assay (Figure S3C) also identified that CUDR overexpression enhanced and CUDR knockdown inhibited the mRNA methylation of SUV39h2 in IL6 treated hepatocyte-like cells. Moreover, RT-PCR findings showed that CUDR overexpression enhanced and CUDR knockdown inhibited SUV39h2 mRNA expression (Fig. 3e). Finally, western blotting and IP results showed that CUDR overexpression enhanced and CUDR knockdown inhibited the expression, phosphorylation and sumoylation of SUV39h2 (Fig. 3f). Collectively, CUDR enhances the expression and modification of SUV39h2 in IL6 treated hepatocyte-like stem cells on the transcriptional and post-transcriptional level (mRNA methylation modification).

### CUDR cooperates with IL6 to enhance expression and phosphorylation of NF-κB through SUV39h2 in the inflammatory environment

**Figure 4:**
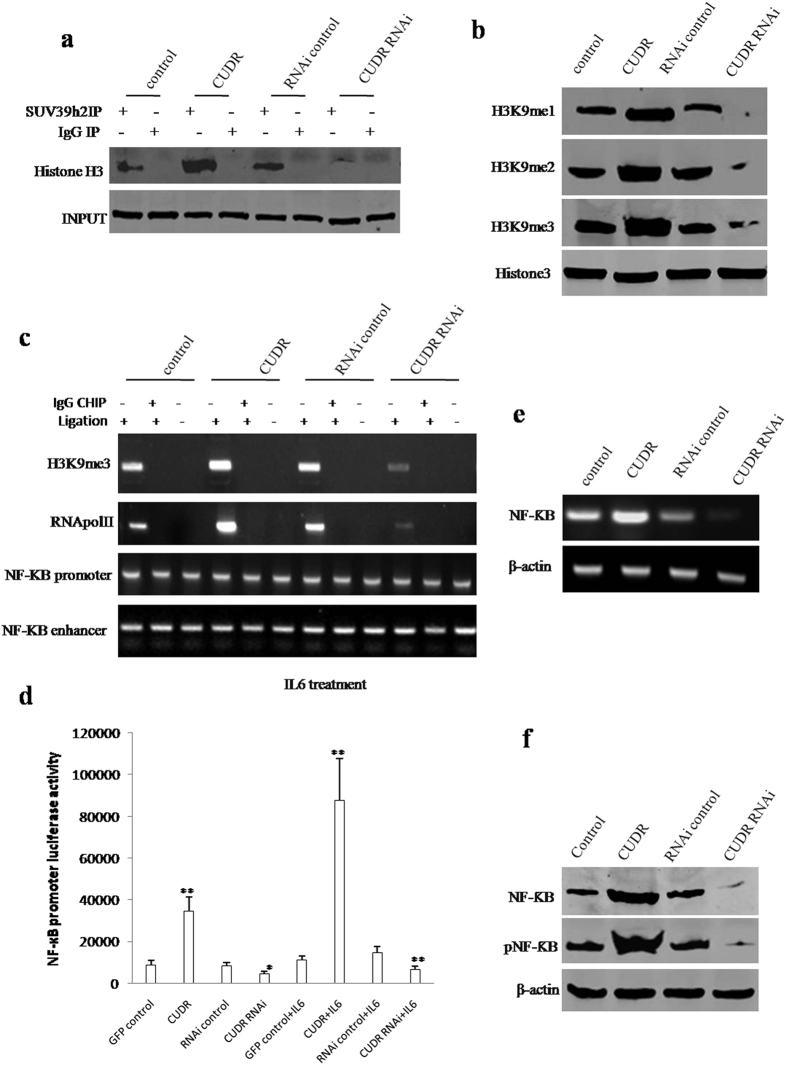
CUDR cooperates with IL6 to enhance the expression and phosphorylation of NF-κB. (**a**) Anti-SUV39h2 **Co-**Immunoprecipitation (IP) followed by western blotting with anti-Histone H3 in hepatocyte-like cellstreated with IL6 and transfected with pCMV6-A-GFP, pCMV6-A-GFP-CUDR, pGFP-V-RS, pGFP-V-RS-CUDR. IgG IP as negative control. INPUT refers to western blotting with anti-Suv39h2. (**b)** Western blotting with anti-H3K9me1, anti-H3K9me2, anti-H3K9me3 in IL6 treated hepatocyte-like cells transfected with pCMV6-A-GFP, pCMV6-A-GFP-CUDR, pGFP-V-RS, pGFP-V-RS-CUDR. Histone3 as internal control. (**c**) Chromosome conformation capture (3C) -chromatin immunoprecipitation (ChIP) with anti-H3K9me3, anti-RNA polII in hepatocyte-like cells treated with IL6 and transfected with pCMV6-A-GFP, pCMV6-A-GFP-CUDR, pGFP-V-RS, pGFP-V-RS-CUDR. The chromatin is cross-linked, digested with restriction enzymes, and ligated under conditions that favor intramolecular ligation. Immediately after ligation, the chromatin is immunoprecipitated using an antibody (anti-H3K9me3, anti-RNA polII) against the protein of interest. Thereafter, the cross-links are reversed, and the DNA is purified further. The PCR anlysis is applied for detecting NF-κB promoter-enhancer coupling product using NF-κB promoter and enhancer primers. The NF-κB promoter and enhancer as INPUT. (**d**) NF-κB promoter luciferase activity assay. Each value was presented as mean ± standard error of the mean (SEM), **P < 0.01. (**e**) The analysis of NF-κB expression by RT-PCR with NF-κB primers. β-actin as internal control. (**f**) Western blotting with anti-NFκB and anti-pNFκB. β-actin as internal control.

Activated NF**κ**B translocates into the nucleus and stimulates the expression of genes involved in a wide variety of biological functions. To validate whether CUDR combined with IL6 could enhance the expression and phorsphorylation of NF-κB, we preformed **Co-**Immunoprecipitation (IP), Western blotting, Chromosome conformation capture (3C)-chromatin immunoprecipitation (ChIP), promoter luciferase activity assay and RT-PCR in IL6 treated hepatocyte-like stem cells transfected with pCMV6-A-GFP, pCMV6-A-GFP-CUDR, pGFP-V-RS, pGFP-V-RS-CUDR. As shown in Fig. 4a, CUDR overexpression promoted and CUDR knockdown inhibited the interplay between SUV39h2 and Histone H3. CUDR overexpression promoted and CUDR knockdown inhibited the modifications of mono-, double-, tri-methylation on the ninth lysine of Histone 3 (H3K9me1, H3K9me2, H3K9me3) (Fig. 4b). Furthermore, CUDR overexpression promoted and CUDR knockdown inhibited NF**κ**B promoter-ehhancer DNA loop formation and blocked the H3K9me3, RNA polymerase II entering into the loop in these IL6 treated hepatocyte-like cells (Fig. 4c). However, this action was abrogated when these hepatocyte-like cells were not treated with IL6 (Figure S4). Moreover, the luciferase activity assay showed that CUDR overexpression promoted and CUDR knockdown inhibited NF**κ**B promoter luciferase activity in these IL6 treated hepatocyte-like cells (Fig. 4d). RT-PCR results showed CUDR overexpression promoted and CUDR knockdown inhibited NF**κ**B transcription in these IL6 treated hepatocyte-like stem cells (Fig. 4e). Western blotting showed CUDR overexpression promoted and CUDR knockdown inhibited the expression and its phosphorylation of NF**κ**B in these IL6 treated hepatocyte-like stem cells (Fig. 4f). Collectively, these observations suggest that CUDR enhances the expression and phosphorylation of NFκB in IL6 treated hepatocyte-like stem cells.

### CUDR plus IL6 enhances the expression and phosphorylation of Stat3 through pNF-κB

**Figure 5:**
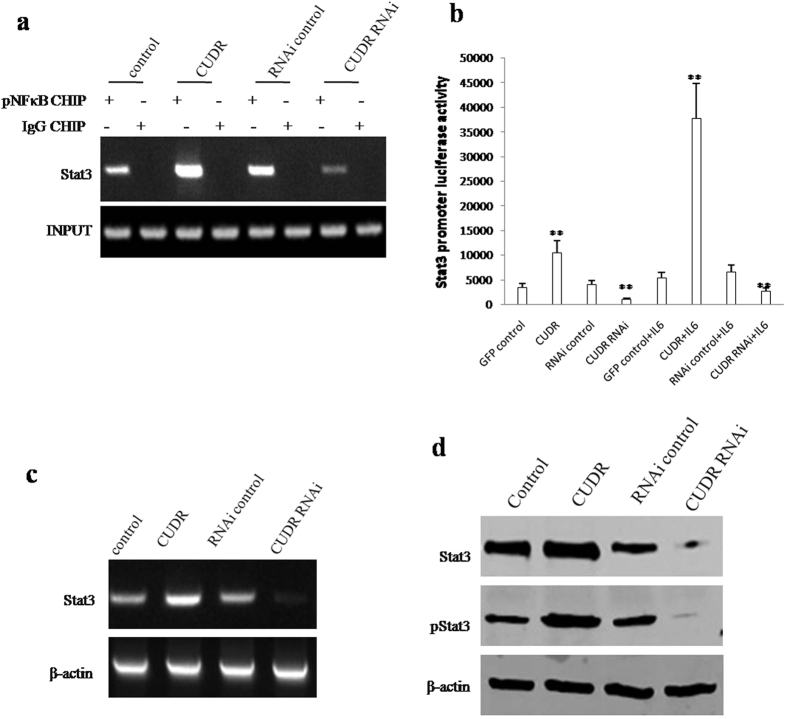
CUDR plus IL6 enhances Stat3 expression and phorsphorylation through pNF-κB. (**a**) Chromatin Immunoprecipitation (CHIP) with anti- pNF-κB followed by PCR with Stat3 promoter primers in hepatocyte-like stem cells treated with IL6 and transfected with pCMV6-A-GFP, pCMV6-A-GFP-CUDR, pGFP-V-RS, pGFP-V-RS-CUDR. IgG CHIP as negative control. Stat3 promoter DNA as INPUT. (**b**) Stat3 promoter luciferase activity assay. Each value was presented as mean ± standard error of the mean (SEM). **P < 0.01. (**c**) The analysis of Stat3 expression by RT-PCR with Stat3 primers. β-actin as internal control. (**d**) Western blotting with anti-Stat3 and pStat3 in hepatocyte-like stem cells treated with IL6 and transfected with pCMV6-A-GFP, pCMV6-A-GFP-CUDR, pGFP-V-RS, pGFP-V-RS-CUDR. β-actin as internal control.

To address whether CUDR altered the expression and its phosphorylation of Stat3 through NFκB, we first performed the Chromatin Immunoprecipitation (CHIP) assay in hepatocyte-like stem cells treated with IL6 and transfected with pCMV6-A-GFP, pCMV6-A-GFP-CUDR, pGFP-V-RS, pGFP-V-RS-CUDR. As show in the Fig. 5a, excessive CUDR enhanced and CUDR knockdown inhibited the pNFκB occupancy on the Stat3 promoter region. However, in hepatocyte-like stem cells untreated with IL6, neither CUDR overexpression nor CUDR knockdown did significantly alter the pNFκB occupancy on the Stat3 promoter region (**data not shown**). Moreover, excessive CUDR increased and CUDR knockdown reduced the Stat3 promoter luciferase activity in hepatocyte-like stem cells treated with IL6 (Fig. 5b). Therefore, excessive CUDR promotes and CUDR knockdown inhibited the transcription (Fig. 5c), translation and phosphorylation (Fig. 5d) of Stat3 in hepatocyte-like cells treated with IL6. Together, these observations suggest that excessive CUDR cooperates with IL6 to enhance the expression and phosphorylation of Stat3 dependent on NF-κB phosphorylation.

### CUDR prompts to alter the expression of microRNAs and lncRNAs through pStat3 in the inflammatory environment

**Figure 6:**
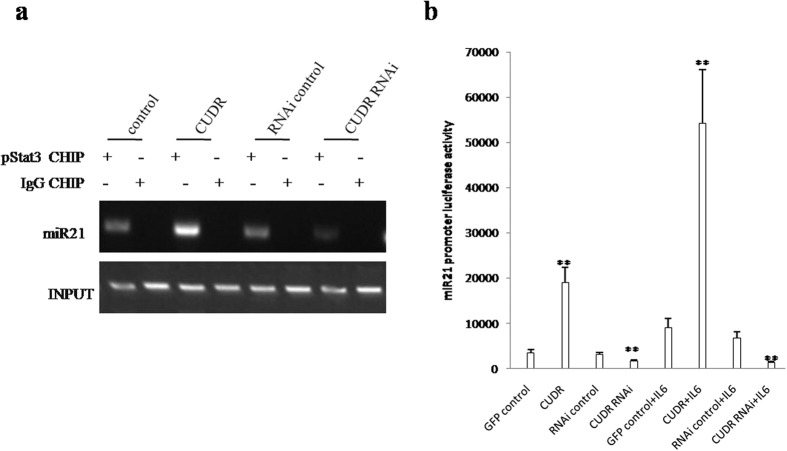
CUDR plus IL6 promotes miRs expression through pStat3. (**a**) Chromatin Immunoprecipitation (CHIP) with anti-pStat3 followed by PCR with miR21 promoter primer in hepatocyte-like cells treated with and IL6 transfected with pCMV6-A-GFP, pCMV6-A-GFP-CUDR, pGFP-V-RS, pGFP-V-RS-CUDR. IgG CHIP as negative control. miR21 promoter DNA as INPUT. (**b**) miR21 promoter luciferase activity assay. Each value was presented as mean ± standard error of the mean (SEM). **P < 0.01.

**Figure 7:**
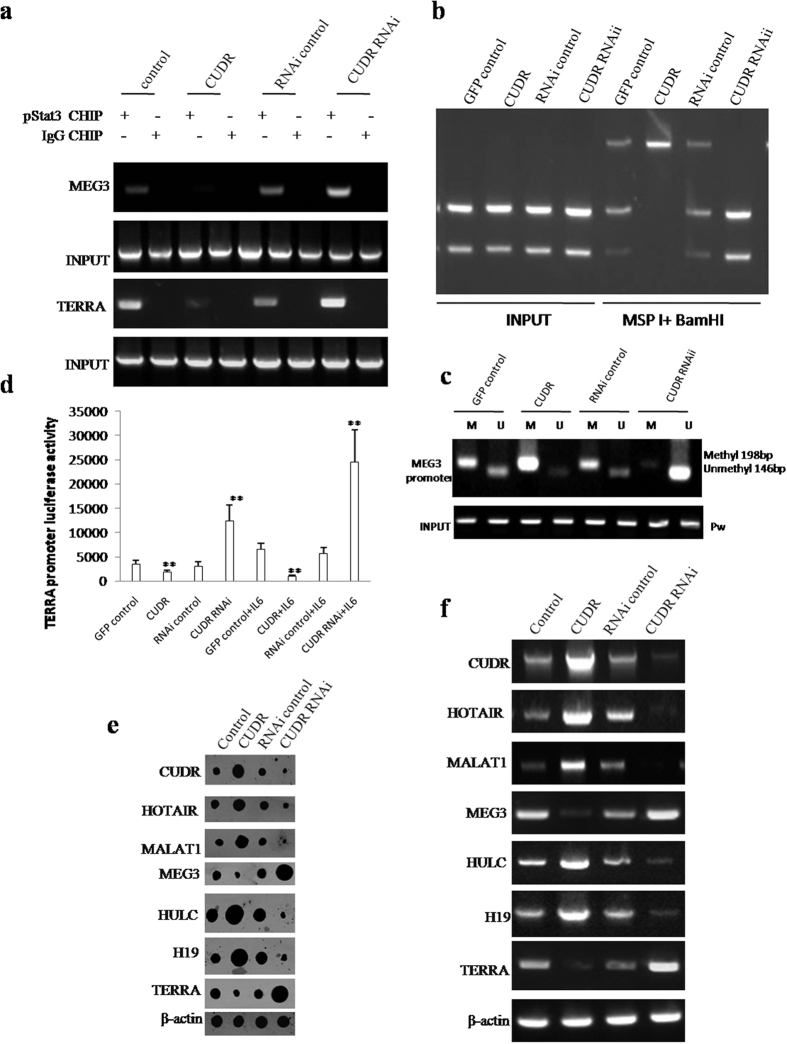
CUDR plus IL6 alters long noncoding RNA expression through pStat3. (**a**) Chromatin Immunoprecipitation (CHIP) with anti- pStat3 followed by PCR with MEG3, TERRA promoter primer in hepatocyte-like cells treated with IL6 and transfected with pCMV6-A-GFP, pCMV6-A-GFP-CUDR, pGFP-V-RS, pGFP-V-RS-CUDR. IgG CHIP as negative control. MEG3, TERRA promoter DNA as INPUT. (**b**). (**a**) TERRA promoter methylation analysis by MspI plus BamHI digestion in hepatocyte-like cells treated with and transfected with pCMV6-A-GFP, pCMV6-A-GFP-CUDR, pGFP-V-RS, pGFP-V-RS-CUDR. (**b)** MEG3 promoter methylation analysis with MSP method primer in hepatocyte-like cells treated with IL6 and transfected with pCMV6-A-GFP, pCMV6-A-GFP-CUDR, pGFP-V-RS, pGFP-V-RS-CUDR. (**c**) CUDR, HOTAIR, MALAT1, MEG3, HULC, H19, TERRA promoter luciferase activity assay. Each value was presented as mean ± standard error of the mean (SEM). **P < 0.01. (**d**) Nuclear run on analysis with Biotin probe of CUDR, HOTAIR, MALAT1, MEG3, HULC, H19, TERRA in hepatocyte-like cells treated with IL6 and transfected with pCMV6-A-GFP, pCMV6-A-GFP-CUDR, pGFP-V-RS, pGFP-V-RS-CUDR. β-actin as internal control. (**e**) RT-PCR analysis with Biotin probe of CUDR, HOTAIR, MALAT1, MEG3, HULC, H19, TERRA in hepatocyte-like stem cells treated with IL6 and transfected with pCMV6-A-GFP, pCMV6-A-GFP-CUDR, pGFP-V-RS, pGFP-V-RS-CUDR. β-actin as internal control.

To elucidate whether CUDR alters the expression of microRNAs and lncRNAs through pStat3 under the inflammatory condition, we first performed Chromatin immunoprecipitation (CHIP) with anti-pStat3 followed by PCR with miR21, miR155, miR17, miR675, miR372, miR192 promoter primers in IL6 treated hepatocyte-like cells transfected with pCMV6-A-GFP, pCMV6-A-GFP-CUDR, pGFP-V-RS, pGFP-V-RS-CUDR. As shown in Figs 6a and S5A, excessive CUDR enhanced and CUDR knockdown inhibited the loading of pStat3 on the promoter region of miR21, miR155, miR17, miR675, miR372, miR192. Excessive CUDR increased and CUDR knockdown decreased the miR21, miR155, miR17, miR675, miR372, miR192 promoter luciferase activity (Fig. 6b). Nuclear run on assay showed that excessive CUDR enhanced and CUDR knockdown inhibited the expression of miR21, miR155, miR17, miR675, miR372, miR192 (Figure S5B). Furthermore, excessive CUDR enhanced and CUDR knockdown inhibited the loading of pStat3 on the promoter region of CUDR, HOTAIR, MALAT1, HULC, H19. Excessive CUDR inhibited and CUDR knockdown enhanced the loading of pStat3 on the promoter region of MEG3, TERRA (Figs 7a and S6). Strikingly, excessive CUDR increased and CUDR knockdown decreased the methylation on promoter regions of TERRA (Fig. 7a) and MEG3 (Fig. 7b). Moreover, excessive CUDR decreased and CUDR knockdown increased the TERRA promoter luciferase activity (Fig. 7c). Furthermore, Nuclear Run-on assay showed that excessive CUDR promoted and CUDR knockdown inhibited the expression of CUDR, HOTAIR, MALAT1, HULC, H19, as well as excessive CUDR inhibited and CUDR knockdown increased the expression of MEG3 and TERRA (Fig. 7d). Moreover, RT-PCR results showed that CUDR promoted and CUDR knockdown inhibited the expression of CUDR, HOTAIR, MALAT1, HULC, H19, as well as excessive CUDR inhibited and CUDR knockdown increased the expression of MEG3 and TERRA (Fig. 7e). Taken Together, these observations suggest that CUDR prompts to alter the expression of microRNAs and lncRNAs dependent on pStat3 in IL6 treated hepatocyte-like stem cells.

### CUDR cooperates with IL6 to enhance telomerase activity, elongate telomere length and increase microsatellite instability (MSI)

**Figure 8:**
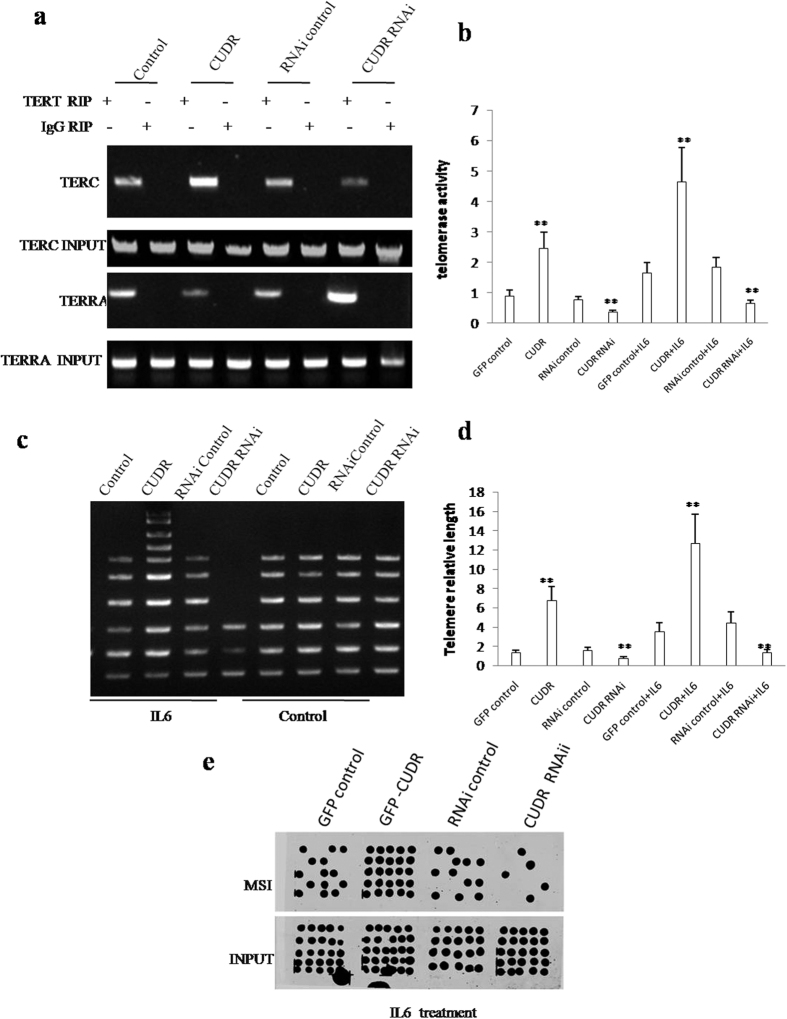
CUDR plus IL6 enhanced telomerase activity, enlongates telomere length and increased Microsatallite Instability (MSI) in CUDR overexpressing hepatocyte-like cells treated with IL6. (**a**) RNA Immunoprecipitation (CHIP) with anti-TERT followed by RT-PCR with TERC and TERRA RNA primers in hepatocyte-like cells treated with IL6 and transfected with pCMV6-A-GFP, pCMV6-A-GFP-CUDR, pGFP-V-RS, pGFP-V-RS-CUDR. IgG CHIP as negative control. TERC and TERRA promoter DNA as INPUT. (**b**) Telomerase activity assay with TRAP method. (**c**) The PCR detection of telomere repeat sequence in IL6 treated hepatocyte-like stem cells transfected with pCMV6-A-GFP, pCMV6-A-GFP-CUDR, pGFP-V-RS, pGFP-V-RS-CUDR. (**d**) The real-time PCR detection of telomere length. Each value was presented as mean ± standard error of the mean (SEM). **P < 0.01. (**e**) Microsatallite Instability (MSI) analysis through Dot blot (Slot blot) using various Biotin labling MSI probes (Biotin-MSIs) in hepatocyte-like cells treated with IL6 and transfected with pCMV6-A-GFP, pCMV6-A-GFP-CUDR, pGFP-V-RS, pGFP-V-RS-CUDR.

Given that CUDR inhibits the TERRA expression which can inhibit TERT activity, we had to consider whether CUDR impacted on telomerase activity, telomere length and microsatellite instability (MSI). In hepatocyte-like cells with IL6 treatment plus CUDR overexpression or depletion, RNA Immunoprecipitation (RIP) with anti-TERT showed that excessive CUDR increased and CUDR knockdown inhibited the interplay between TERT and TERC, as well as excessive CUDR inhibited and CUDR knockedown increased the interplay between TERT and TERRA (Fig. 8a). Excessive CUDR increased and CUDR knockdown inhibited the telomerase activity in hepatocyte-like stem cells with IL6 treatment. However, both excessive CUDR and CUDR knockdown did significantly not alter the telomerase activity in hepatocyte-like stem cells without IL6 treatment (Fig. 8b). The regular PCR detection of telomere repeat sequence in hepatocyte-like cells with IL6 treatment showed that excessive CUDR increased and CUDR knockdown decreased the telomere length. However, this action was abrogated in control group (Fig. 8c). The real-time PCR detection of telomere repeat sequence in hepatocyte-like stem cells with IL6 treatment showed that excessive CUDR increased and CUDR knockdown decreased the telomere length. However, this action was abrogated in control group. However, both excessive CUDR and CUDR knockdown did significantly alter the telomerase length in hepatocyte-like stem cells without IL6 treatment (Fig. 8d). Intriguingly, our results showed that excessive CUDR increased and CUDR knockdown decreased the microsatellite instability(MSI) in hepatocyte-like stem cells with IL6 treatment (Fig. 8e). Together, CUDR enhances telomerase activity, elongates telomere length and increases microsatellite instability(MSI) in the inflammatory environment through miRs or lncRNAs.

### IL6 cooperates with CUDR to aggravate hepatocyte-like stem cells malignant transformation through NF-κB signaling

**Figure 9:**
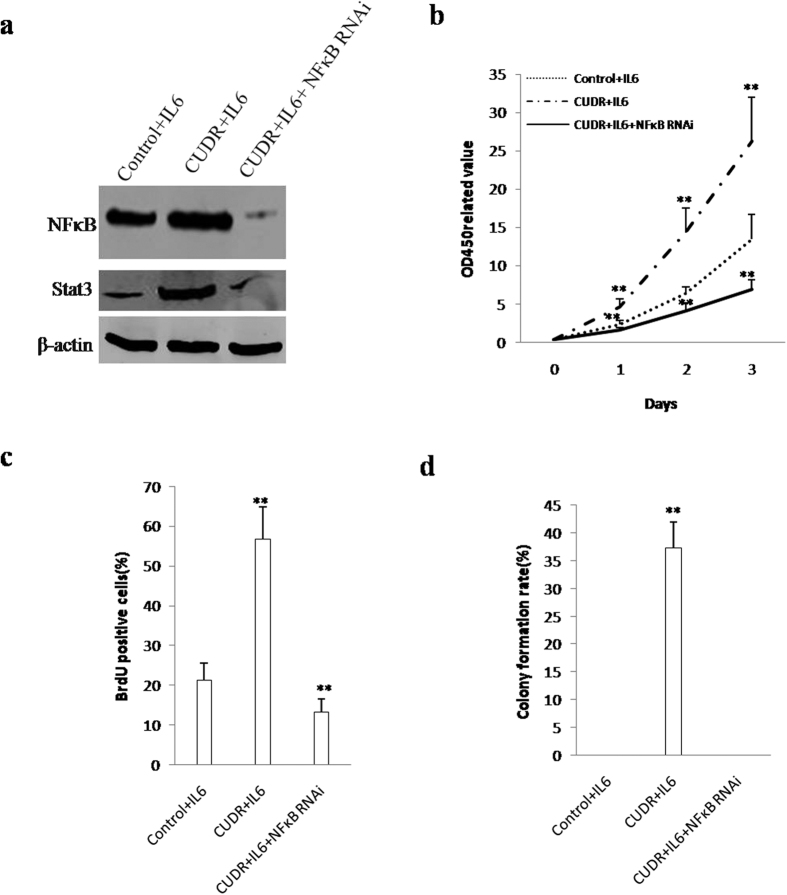
IL6 cooperates with CUDR to aggravate hepatocyte-like stem cells malignant transformation through NF-κB signaling. (**a**) Western blotting analysis with anti- NF-κB, anti-Stat3 in the hepatocytes-like stem cells. β-actin as internal control. (**b**) Cells growth assay using CCK8. Each value was presented as mean ± standard error of the mean (SEM). **P < 0.01. (**c**) BrdU assay in the hepatocytes-like stem cells. Each value was presented as mean ± standard error of the mean (SEM). **P < 0.01. (**d**) Cells soft agar colony formation assay. Each value was presented as mean ± standard error of the mean (SEM). **P < 0.01.

To address whether IL6 cooperates with CUDR to aggravate malignant transformation of hepatocyte-like stem cells through NF-κB signaling, we established hepatocyte-like stem cell lines with CUDR overexpression plus IL6, or CUDR overexpression plus IL6 plus NF-κB knockdown. As shown in Fig. 9a, CUDR overexpression plus IL6 significantly increased the NF-κB and Stat3 expression, howover, CUDR overexpression plus IL6 plus NF-κB knockdown significantly decreased the expression of NF-κB and Stat3 in hepatocyte-like stem cells. As shown in Fig. 9b, excessive CUDR plus IL6 significantly promoted the cell growth of hepatocyte-like stem cells (P < 0,01), however, excessive CUDR combined with IL6 and NF-κB knockdown did not alter cell growth ability (P>0.05). As shown in Fig. 9c, excessive CUDR plus IL6 significantly increased the BrdU positive rate (56.72 ± 8.23% vs 21.317 ± 4.24%, P < 0.01) of hepatocyte-like stem cells, however, CUDR overexpression plus IL6 plus NF-κB knockdown significantly decreased the BrdU positive rate (13.22 ± 3.43% vs 21.317 ± 4.24%, P < 0.01) (P < 0.01). Furthermore, CUDR plus IL6 overexpression significantly increased the colony formation ability (17.3 ± 4.67% vs 0, P < 0.01) of hepatocyte-like stem cells. However, depletion of NFκB fully abrogated the action of CIUDR plus IL6 (Fig. 9d). Taken Together, these observations suggest that IL6 cooperates with CUDR to aggravate malignant transformation of hepatocyte-like stem cells through NF-κB signaling.

## Discussion

**Figure 10:**
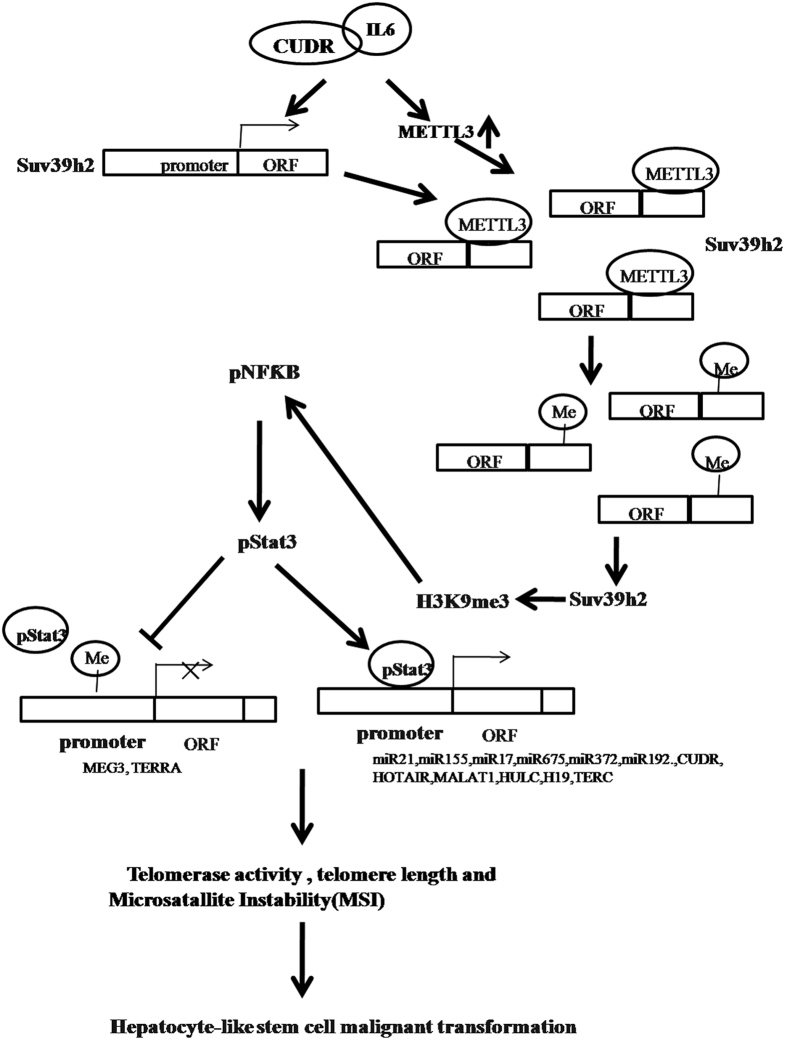
The schematic diagram illustrates a model that IL6 cooperates with CUDR to promote malignant transformation of hepatocyte-like stem cells. The interaction between CUDR and inflammatory cytokine IL6 triggers the malignant transformation of human embryonic stem cells derived hepatocyte-like stem cells. Mechanistically, our results reveal CUDR cooperates with IL6 to promote SUV39h1 high expression at the transcriptional level in the inflammatory environment. Intriguingly, CUDR cooperates with IL6 also to cause METTL3 overexpression that bind to SUV39h1 mRNA. Accordingly, the methylation of SUV39h1 mRNA 3 ‘UTR is increased that causes the SUV39h1mRNA more stable. Then, the excessive SUV39h1 increases three methylation on histone H3 nineth lysine (H3K9me3). In inflammatory conditions, H3K9me3 promotes the expression and phosphorylation of NF-κB, and phorsphorylated NF-κB promotes the expression and phosphorylation of Stat3. That phosphorylated Stat3 loads onto the promoter region of miR21, miR155, miR17, miR675, miR372, miR192, CUDR, HOTAIR, MALAT1, HULC, H19 enhances these noncoding RNAs outcome. On the other hand, excessive CUDR and IL6 increases DNA methylation of MEG3, TERRA promoter region. Therefore, the binding of pStat3 to MEG3, TERRA the promoter regions is decreased that leads to reduce the MEG3, TERRA expression. Ultimately, the abnormal expression of miRs and lncRNAs results in increased telomerase activity, telomere length and the increased microsatallite instability (MSI), eventually leading to malignant transformation of hepatocyte-like stem cells.

Long nocoding RNA (LncRNAs) can regulate gene expression in many ways, including chromosome remodeling, transcription and post-transcriptional processing. Moreover, the dysregulation of lncRNAs has increasingly been linked to many human diseases, especially in cancers^30^. To this data, we revealed that CUDR cooperates with inflammatory cytokine IL6 to triggers the malignant transformation of human embryonic stem cells derived hepatocyte-like stem cells. Mechanistically, our results demonstrate IL6 cooperates with CUDR to promote SUV39h1 expression in the inflammatory environment. Accordingly, the methylation of SUV39h1 mRNA 3′ UTR is increased that causes the SUV39h1 mRNA more stable. Furthermore, the excessive SUV39h1 increases three methylation on histone H3 ninth lysine (H3K9me3). Under inflammatory conditions, H3K9me3 promotes the expression and phosphorylation of NF-κB, in turn, phorsphorylated NF-κB promotes the expression and phosphorylation of Stat3. Importantly, the phosphorylated Stat3 loads onto the promoter region of miR21, miR155, miR17, miR675, miR372, miR192, CUDR, HOTAIR, MALAT1, HULC, H19, as well as excessive CUDR and IL6 increases DNA methylation of MEG3, TERRA promoter region. Therefore, the binding of pStat3 to MEG3, TERRA the promoter regions is decreased that leads to reduce the expression of MEG3, TERRA. Ultimately, the abnormal expression of miRs and lncRNAs results in increased telomerase activity, telomere length and the increased microsatallite instability (MSI), leading to malignant transformation of hepatocyte-like cells eventually (Fig. 10).

Interleukin-6 could induced malignant transformation of stem cells in association with enhanced signaling of Stat3^31^ and myeloid-derived cells endowed stem-like qualities to breast cancer cells through IL6/STAT3 and NO/NOTCH cross-talk signaling^32^. In particular, IL6 blockade significantly inhibited lung cancer promotion^33^. Our results showed that IL6 alone could not prompt malignant transformation of hepatocyte-like stem cells, however, IL6 enhanced the CUDR oncogenic action that triggered malignant transformation of hepatocyte-like stem cells. It suggests IL6 acts as enhancer of tumorigenesis. To our knowledge, this is the first report demonstrating CUDR cooperates with IL6 to trigger malignant transformation of hepatocyte-like cells *in vitro* and *in vivo*.

It is worth mentioning that CUDR plus IL6 may play an important role in the occurrence of hepatocellular carcinoma. In this report, we focused mainly on the view how CUDR plus IL6 functions during hepatocyte-like stem cells’ malignant transformation. To this date, accumulating evidence indicates that CUDR was aberrantly upregulated in liver cancer tissues and associated with TNM stage, metastasis and postoperative survival. CUDR depletion inhibited the growth and metastasis of HCC cell lines *in vitro* and *in vivo*. For example, excessive CUDR contributes to progression of hepatocellular carcinoma through inhibition of miR-216b and activation of FGFR1/ERK signaling pathway^34^. Our present findings are consistent with some reports. Overexpression of the CUDR plus IL6 links a dramatic increase of proliferative and growth capacity. Herein, the involvement of promotion of hepatocyte-like stem cells growth based on CUDR plus IL6 is supported by results from four parallel sets of experiments: 1) excessive CUDR triggers malignant transformation of human embryonic stem cells derived hepatocyte-like stem cells in mouse injury liver inflammation microenvironment; 2) excessive CUDR cooperates with IL6 to aggravate derived hepatocyte-like cells malignant transformation and growth of hepatocyte-like cells *in vitro* and *in vivo*. 3) In IL6 treated groups, excessive CUDR enhanced the mPGES1, TLR4, NF-κB expression, as well as reduced the 15PGDH expression in these induced hepatocyte-like cells. On the other hand, mPGES1, TLR4, NF-κB, 15PGDH expression were significantly not changed in IL6 untreated groups. According to the aforementioned findings and reports, it is clear that CUDR plus IL6 has a strong carcinogenic ability.

It has been confirmed that histone H3 lysine 9 trimethylation (H3K9me3) is associated with transcriptional repression and regulated by histone lysine methyltransferases such as suppressor of variegation 3–9 homolog 1 (SUV39H1), a histone methyltransferase. SUV39H1 catalyzes histone 3 lysine 9 trimethylation and is involved in heterochromatin organization and genome stability. Methylation of SUV39H1 facilitated genome instability and ultimately inhibited cell proliferation. SUV39H1 knockdown reduced H3K9me3 levels and impaired HCC cell growth and sphere formation. Elevated SUV39H1 expression and high levels of H3K9me3 have important roles in HCC development and progression^35^,^36^. The sustained expression of SUV39H1 delays the repair of constitutive heterochromatin (HC) DNA and reduces clonogenic survival after ionizing irradiation^37^. Moreover, long H3K9me3-marked domains had lower accessibility, consistent with a more compact chromatin structure^38^. Dynamic changes in histone modification are critical for regulating DNA double-strand break (DSB) repair, increasing the levels of H3K9me3 in open chromatin domains that lack H3K9me3 and thereby promoting efficient activation of Tip60 and ATM in these regions^39^. On the other hand, METTL3, an N(6)-methyladenosine (m(6)A) transferase, as a regulator for terminating murine naïve pluripotency. METTL3 knockout preimplantation epiblasts and naïve embryonic stem cells are depleted for m(6)A in mRNAs^40^ and N6-methyladenosine modification destabilizes developmental regulators in embryonic stem cells^41^. RNA decoration by m(6)A has a fundamental role in regulation of gene expression^42^. Our data suggest that CUDR enhances SUV39h2 expression and H3K9me3 modification dependent on IL6 on the transcriptional and post-transcriptional mRNA methylation modification level. This assertion is based on several observations in IL6 treated hepatocyte-like stem cells: (1) CUDR facilitated the loading of CREPT (***c***ell-cycle ***r***elated and ***e***xpression- elevated ***p***rotein in ***t***umor) and RNA polymerase II onto SUV39h2 promoter region. (2) CUDR increased the SUV39h2 promoter luciferase activity. (3) CUDR enhanced the METTL3 expression. (4) CUDR enhanced the binding of METTL3 to SUV39h2 mRNA; (5) CUDR enhanced the mRNA methylation of SUV39h2; (6) CUDR enhanced SUV39h2 mRNA expression; (7) CUDR enhanced SUV39h2 expression, phosphorylation and sumoylation. (8) CUDR enhanced three methylation on histone H3 nineth lysine (H3K9me3).

Sumoylation belongs to the host cell posttranslational modification system which is an enzymatic process that is biochemically analogous to, but functionally distinct from, ubiquitinylation. Conjugation of SUMO does not typically lead to degradation of the substrate. It is clear that this modification system is an important regulator of intracellular protein localization, particularly involving nuclear uptake and punctate intranuclear accumulation^43^,^44^. In the report, our findings showed that CUDR enhanced SUV39h2 expression through sumoylation.

FTO gene encods an N(6)-methyladenosine (m(6)A) RNA demethylase. The most abundant mRNA post-transcriptional modification is N(6)-methyladenosine (m(6)A), which has broad roles in RNA biology by limiting the m(6)A ‘eraser’ FTO from demethylation^45^,^46^. However, CUDR overexpression or knockdown did not alter the FTO expression in IL6 treated hepatocyte-like cells. It suggests CUDR combined with IL6 could enhance RNA methylase activity, however, did not alter RNA demethylase in hepatocyte-like cells.

NF**κ**B has been associated with a number of inflammatory diseases while persistent inhibition of NF**κ**B leads to inappropriate immune cell development or delayed cell growth^47^,^48^. To this data, Our observations suggest that CUDR plus IL6 enhances expression and phosphorylation of NF-κB through SUV39h2 under the inflammatory condition. This assertion is based on several observations: 1) CUDR promoted the interplay between SUV39h2 and HistoneH3. Therefore, excessive CUDR promoted the modifications of mono-, double-, tri-methylation on the ninth lysine of Histone H 3 (H3K9me1, H3K9me2, H3K9me3); 2) CUDR promoted NF**κ**B promoter-ehhancer looping formation and the H3K9me3, RNA polymerase II entering into the loop in these hepatocyte-like cells treated with IL6; 3) CUDR promoted NF**κ**B promoter luciferase activity in these hepatocyte-like cells treated with IL6. 4) excessive CUDR promoted expression and its phosphorylation of NF**κ**B in these hepatocyte-like stem cells treated with IL6. Inaword, these findings are noteworthy that CUDR plus IL6 links to cancer cells growth in the inflammatory environment.

It is worth noting that Stat3 activity alteration is absorbing and of great concern. STATs can be activated independently of JAKs, most notably by c-Src kinases. Several approaches have been used to inhibit STAT3 in the hope of developing an antitumor agent^49^,^50^. Strikingly, STAT3 regulates the migration and invasion of a stem-like subpopulation through microRNA-21 and multiple targets in hepatocellular carcinoma^51^. We observed that excessive CUDR cooperates with IL6 to enhance expression and phosphorylation of Stat3 dependent on NF-κB phosphorylation. It is evident that activation of Stat3 may play an important role in hepatocyte-like cells malignant transformation. Our findings in this study provide novel evidence for an active role of Stat3 promotion of liver cancer stem cell growth. This assertion is based on several observations: 1) CUDR enhanced the pNFκB occupancy on the Stat3 promoter region. 2) CUDR increased the Stat3 promoter luciferase activity in hepatocyte-like cells treated with IL6. 3) CUDR promotes the Stat3 transcription, translation and its phosphorylation in hepatocyte-like cells without treated with IL6.

Strikingly, our results show CUDR plus IL6 alters microRNAs and lncRNAs expression through pStat3 in hepatocyte-like cells. Herein, our findings show that CUDR alters microRNAs and lncRNAs expression through pStat3 in the inflammatory environment dependent on IL6. The conclusion is supported by results from nine parallel sets of experiments: 1) CUDR enhanced the loading of pStat3 on the promoter region of miR21, miR155, miR17, miR675, miR372, miR192. 2) CUDR overexpression increased the miR21, miR155, miR17, miR675, miR372, miR192 promoter luciferase activity. 3) CUDR overexpression enhanced the expression of miR21, miR155, miR17, miR675, miR372, miR192; 4) CUDR enhanced the loading of pStat3 on the promoter region of CUDR, HOTAIR, MALAT1, HULC, H19. 5) CUDR inhibited the loading of pStat3 on the promoter region of MEG3, TERRA; 6) CUDR increased the TERRA and MEG3 methylation on promoter region. 7) CUDR decreased the TERRA promoter luciferase activity; 8) CUDR promoted the expression CUDR, HOTAIR, MALAT1, HULC, H19, as well as CUDR inhibited the expression MEG3 and TERRA; 9) CUDR promoted the expression HOTAIR, MALAT1, HULC H19, as well as CUDR inhibited the expression MEG3 and TERRA.

It is well known that DNA mismatch repair (MMR) ensures replication fidelity by correcting mismatches generated during DNA replication^52^. Proper telomeric chromatin configuration is thought to be essential for telomere homeostasis and stability^53^. The human telomerase reverse transcriptase catalytic subunit (TERT) contributes to cell physiology independently of its ability to elongate telomeres^54^. Telomeres have been considered to be silent, but it was recently demonstrated that mammalian telomeres are transcribed into telomeric repeat-containing RNA (TERRA). TERRA participates in the regulation of telomere length, telomerase activity and heterochromatinization^55^. Our results showed that CUDR cooperated with IL6 to enhance telomerase activity, elongate telomere length and increase Microsatellite Instability(MSI). This assertion is based on several observations: in IL6 treated hepatocyte-like stem cells, 1) CUDR increased the interplay between TERT and TERC, as well as CUDR overexpression inhibited the interaction between TERT and TERRA; 2) excessive CUDR increased the telomerase activity in IL6 treated hepatocyte-like cells; 3) excessive CUDR increased the telomere length; 4) excessive CUDR increased the telomere length; 5) excessive CUDR increased the Microsatellite Instability(MSI) in IL6 treated hepatocyte-like cells.

Many questions remained about the function of CUDR combined with inflammatory factor. For example, what causes strong oncogenic action CUDR plus IL6 inflammatory factor? How does CUDR cooperates with inflammatory factor? Does CUDR enhance malignant differentiation of liver stem cells? Does CUDR regulate a series of molecular events during malignant differentiation of liver stem cells? Answering these questions will help understand the mechanism about malignant differentiation of liver stem cell. **In summary**, our present data indicated that CUDR combined with inflammatory factor promotes malignant progression of liver stem cells through epigenetic hallmark. These observations provide insight into a novel link between CUDR and inflammatory cytokines during hepatocarcinogenesis. To understand the novel functions of CUDR will help in the development of new liver cancer therapeutic and diagnostic approaches.

## Experimental Procedures

### Ethics statement

All methods were carried out in “accordance” with the approved guidelines. All experimental protocols “were approved by” a Tongji university institutional committee. Informed consent was obtained from all subjects. The study was reviewed and approved by the China national institutional animal care and use committee”.

### Cell Lines

Human embryonic stem (ES) cells line MEL-2 (Merck Millipore, Darmstadt, Germany) were maintained in HEScGRO Medium (1000IU/ml LIF) (Merck Millipore, Darmstadt, Germany) supplemented with 10% heat-inactivated fetal bovine serum (Gibco BRL Life Technologies, Grand Island, NY) on matrigel (0.1% gelatin solution or human collagen IV coating material) in a humidified atmosphere of 5% CO_2_ incubator at 37 °C.

### Intravenous transplantation of the hepatocyte-like stem cells

Viable hepatoblasts (10^8^ cells/mouse) were inoculated i.v. into Balb/C mice. The Balb/C mice were observed over 8 weeks for tumor formation. Animals were stratified so that the mean tumor sizes in all groups were nearly identical. The mice were then sacrificed and the tumors recovered.

### Cell transfection and stable cell lines

Cells were transfected with DNA plasmids using transfast transfection reagent lipofectamine^R^ 2000 (Invitrogen) according to manufacturer’s instructions.

### RT-PCR

Total RNA was purified using Trizol (Invitrogen) according to manufacturer’s instructions. cDNA was prepared by using oligonucleotide (dT)_17–18_, random primers, and a SuperScript First-Strand Synthesis System (Invitrogen). PCR analysis was performed under the specical conditions. β-actin was used as an internal control.

### Co-immunoprecipitation (IP)

Cells were lysed in 1 ml of the whole-cell extract buffer A (50 mM pH7.6 Tris-HCl, 150 mM NaCl, 1%NP40, 0.1 mMEDTA, 1.0 mM DTT, 0.2 mMPMSF, 0.1 mM Pepstatine, 0.1 mM Leupeptine, 0.1 mM Aproine). Five-hundred-microliter cell lysates was used in immunoprecipitation with antibody. In brief, protein was pre-cleared with 30 μl protein G/A-plus agarose beads (Santa Cruz, Biotechnology, Inc. CA) for 1 hour at 4 °C and the supernatant was obtained after centrifugation (5,000 rpm) at 4 °C. Precleared homogenates (supernatant) were incubated with 2 μg of antibody and/or normal mouse/rabbit IgG by rotation for 4 hours at 4 °C, and then the immunoprecipitates were incubated with 30 μl protein G/A-plus agarose beads by rotation overnight at 4 °C, and then centrifuged at 5000 rpm for 5 min at 4 °C. The precipitates were washed five times ×10 min with beads wash solution (50 mM pH7.6 TrisCl, 150 mMNaCl, 0.1%NP-40, 1 mM EDTA) and then resuspended in 60 μl 2 × SDS-PAGE sample loading buffer to incubate for 5–10 min at 100 °C. Then Western blot was performed with a another related antibody indicated in Western blotting.

### Chromatin immunoprecipitation (CHIP)

Cells were cross-linked with 1% (v/v) formaldehyde (Sigma) for 10 min at room temperature and stopped with 125 mM glycine for 5 min. Crossed-linked cells were washed with phosphate-buffered saline, resuspended in lysis buffer, and sonicated for 8–10 min in a SONICS VibraCell to generate DNA fragments with an average size of 500 bp or so. Chromatin extracts were diluted 5-fold with dilution buffer, pre-cleared with Protein-A/G-Sepharose beads, and immunoprecipitated with specific antibody on Protein-A/G-Sepharose beads. After washing, elution and de-cross-linking, the ChIP DNA was detected by either traditional PCR.

### Chromosome conformation capture (3C)-CHIP

Antibody-specific immunoprecipitated chromatin was obtained as described above for ChIP assays. PCR products were run on a 2% agarose gel. Each validation experiment was repeated at least twice.

### Methylation analysis

Mthylated DNA Immunoprecipitation (MeDIP)-Dot blot-western blotting with anti-5-Methylcytosine (5-mC) and ethylation analysis by MspI plus BamHI digestion.

### Soft agar colony formation assay

2 × 10^2^ cells were plated on a 6 well plate containing 0.5% (lower) and 0.35% (upper) double layer soft-agar. The cells in six well plates were incubated at 37 °C in humidified incubator for 21 days. The cells were fed 1–2 times per week with cell culture media (DMEM). Soft-agar colonies on the 6 well plates were stained with 0.5 ml of 0.05% Crystal Violet for more than 1 hour and the colonies were counted.

### Statistical analysis

The significant differences between mean values obtained from at least three independent experiments. Each value was presented as mean ± standard error of the mean (SEM) unless otherwise noted, with a minimum of three replicates. Student’s t-test was used for comparisons, with P < 0.05 considered significant.

## Additional Information

**How to cite this article**: Zheng, Q. *et al*. Inflammatory cytokine IL6 cooperates with CUDR to aggravate hepatocyte-like stem cells malignant transformation through NF-κB signaling. *Sci. Rep*. **6**, 36843; doi: 10.1038/srep36843 (2016).

**Publisher’s note**: Springer Nature remains neutral with regard to jurisdictional claims in published maps and institutional affiliations.

## Supplementary Material

Supplementary Information
