# Supplementary material for: Inflammatory cytokine IL6 cooperates with CUDR to aggravate hepatocyte-like stem cells malignant transformation through NF-κB signaling
Source: Sci Rep. 2016 Nov 11;6:36843. doi: 10.1038/srep36843 (PMC5104983; doi:10.1038/srep36843)
Supplement: Supplementary Information [file srep36843-s1.pdf]

supplementary information

Inflammatory cytokine IL6 cooperates with CUDR to aggravate hepatocyte-like stem cells malignant transformation through NF-κB signaling

Qidi Zheng[1], Zhuojia Lin[1], Xiaonan Li[1], Xiaoru Xin[1],
Mengying Wu[1],
Jiahui An[1],
Xin Gui[1],
Tianming Li[1],
Hu Pu[1],
Haiyan Li[1] ,
Dongdong Lu [1,*]

[1.]School of Life Science and Technology, Tongji University ， Shanghai(200092), China

* Corresponding author: *Dongdong Lu*, Tongji University School of Life Science and Technology, Shanghai(200092), China.　E-mail: *ludongdong@tongji.edu.cn*

supplementary　Experimental Procedures

Ethics statement All methods were carried out in "accordance" with the approved guidelines. All experimental protocols "were approved by" a Tongji university institutional committee. Informed consent was obtained from all subjects. The study was reviewed and approved by the China national institutional animal care and use committee".

Cell Lines and Plasmids Human embryonic stem (ES) cells line MEL-2(Merck Millipore, Darmstadt, Germany) were maintained in HEScGRO Medium(**1000IU/ml** LIF)**(**Merck Millipore, Darmstadt, Germany) supplemented with 10% heat-inactivated fetal bovine serum (Gibco BRL Life Technologies ,Grand Island,NY) on matrigel (0.1 % gelatin solution or human collagen IV coating material) in a humidified atmosphere of 5% CO_2 incubator at 37ºC. Mitocally inactivated HS27 feeder cells (ATCC,Manassas,VA) plated at 80,000 cells/cm^2. Plasmid pGFP-V-RS, pCMV6-A-GFP were purchased from Origene (Rockville, MD 20850,USA).pGL3-NFκB promoter, pGL3-Stat3 promoter were purchased from Addgene(Cambridge MA,USA).pGL3-miR21 promoter, pGL3-miR155 promoter , pGL3-miR17 promoter , pGL3-miR675 promoter, pGL3-miR372 promoter , pGL3-miR192 promoter CUDR, pGL3-HOTAIR promoter, pGL3-MALAT1-promoter, pGL3-MEG3 promoter , pGL3-HULC promoter, pGL3-H19 promoter, pGL3-TERRA promoter, pGL3-TERC promoter pGFP-V-RS-CUDR, pCMV6-A-CUDR were constructed by ourselves.

Embryonic stem (ES) cells differentiate into hepatocyte-like cell in vitro Human ES cell line MEL-2 could efficiently generate definitive endoderm (DE) tissue by treating the modified cultures with high concentrations of the TGFβ family ligand activin A(100 ng/ml, R&D, Minneapolis) for 5 days. A number of groups have generated hepatoblosts using this DE tissue as a starting material, plating the DE on matrix (e.g. collagen) to mimic the hepatic ECM and then added FGF4(100 ng/ml,R&D) and BMP(100 ng/ml ,R&D, Minneapolis) to mimic hepatic induction for 6 days (induced hepatoblasts). This is followed by some combination of insulin, transferrin, selenite(ITS,5μg/ml, R&D, Minneapolis), HGF(20ng/ml, R&D, Minneapolis),OSM(10ng/ml, R&D, Minneapolis),aFGF(50ng/ml, R&D, Minneapolis)

and Dexamethasone(10^{-7}M, R&D, Minneapolis) to expand the hepatoblasts population and to promote hepatic maturation for 10 days**.**

Mouse Liver *In Vivo* Transfection Systemic administration (i.v. Final injection volume 0.5ml) of Balb/C mouse (20-24g) using Liver *In Vivo* Transfection Reagent (Altogen Biosystems, Las Vegas, NV 89107,USA) conjugated with plasmid DNA. In brief, first, dilute 60 μg of plasmid DNA in 100 μl RNase-/DNase-free water vortex gently. Secondly, add 100 μl of diluted DNA to the sterile tube containing 50 μl Transfection Reagent and incubate for 15-20 minutes at room temperature. Thirdly, add 10 μl of Transfection Enhancer Reagent vortex gently and incubate for 5 minutes at room temperature. Then add required amount of sterile solution of 5% glucose (w/v) for injecting animals. The delivery efficiency can be significantly increased by performing repeat injection at least 12 hours after first injection till stable intergration. The use of mice for this work was reviewed and approved by the institutional animal care and use committee in accordance with China national institutes of health guidelines.

RT-PCR Total RNA was purified using Trizol (Invitrogen) according to manufacturer's instructions. cDNA was prepared by using oligonucleotide $(dT)_{17\text{-}18}$, random primers, and a SuperScript First-Strand Synthesis System (Invitrogen). PCR analysis was performed under the specical conditions. β-actin was used as an internal control.

Western Blotting The logarithmically growing cells were washed twice with ice-cold phosphate-buffered saline (PBS, Hyclone) and lysed in a RIPA lysis buffer. Cells lysates were centrifuged at 12,000g for 20 minutes at 4°C after sonication on ice , and the supernatant were separated. After being boiled for 5-10 minutes in the presence of 2-mercaptoethanol, samples containing cells proteins were separated on a 10% sodium dodecyl sulfate-polyacrylamide gel electrophoresis (SDS-PAGE) and transferred onto a nitrocellulose membranes (Invitrogen, Carlsbad, CA,USA). Then blocked in 10% dry milk-TBST (20mM Tris-HCl [PH 7.6], 127mM NaCl, 0.1% Tween 20) for 1 h at 37°C. Following three washes in Tris-HCl pH 7.5 with 0.1% Tween 20, the blots were incubated with 0.2 μg/ml of antibody(appropriate dilution)

overnight at 4°C. Following three washes, membranes were then incubated with secondary antibody for 60 min at 37°C or 4°C overnight in TBST. Signals were visualized by ECL.

Co-immunoprecipitation(IP) Cells were lysed in 1 ml of the whole-cell extract buffer A(50mM pH7.6 Tris-HCl, 150mM NaCl, 1%NP40, 0.1mMEDTA,1.0mM DTT,0.2mMPMSF, 0.1mM Pepstatine,0.1mM Leupeptine,0.1mM Aproine). Five-hundred-microliter cell lysates was used in immunoprecipitation with antibody. In brief, protein was pre-cleared with 30μl protein G/A-plus agarose beads (Santa Cruz, Biotechnology,Inc.CA) for 1 hour at 4°C and the supernatant was obtained after centrifugation (5,000rpm) at 4°C. Precleared homogenates(supernatant) were incubated with 2 μg of antibody and/or normal mouse/rabbit IgG by rotation for 4 hours at 4°C,and then the immunoprecipitates were incubated with 30μl protein G/A-plus agarose beads by rotation overnight at 4°C,and then centrifuged at 5000rpm for 5 min at 4°C. The precipitates were washed five times×10min with beads wash solution(50 mM pH7.6 TrisCl,150mMNaCl,0.1%NP-40,1mM EDTA) and then resuspended in 60μl 2×SDS-PAGE sample loading buffer to incubate for 5-10 min at 100°C. Then Western blot was performed with a another related antibody indicated in Western blotting.

RNA Immunoprecipitation(RIP) Cells were lysed (15 min, 0°C) in 100 mM KCl, 5 mM $MgCl_2$, 10 mM HEPES [pH 7.0], 0.5% NP40, 1 mM DTT, 100 units/ml RNase OUT (Invitrogen), 400 μM vanadyl-ribonucleoside complex and protease inhibitors (Roche), clarified and stored on at −80°C. Ribonucleoprotein particle-enriched lysates were incubated with protein A/G-plus agarose beads (Santa Cruz, Biotechnology, Inc.CA) together with antibody or normal mouse or rabbit IgG for 4 hours at 4°C. Beads were subsequently washed four times with 50 mM Tris-HCl(pH 7.0), 150 mM NaCl, 1 mM $MgCl_2$, and 0.05% NP-40, and twice after addition of 1M Urea. Immunoprecipitates(IPs) were digested with proteinase K (55°C; 30′) and mRNAs were then isolated and purified. RT-PCR was performed with the primers as follows: CUDR/P1:5’-atgagtcccatcatctctcca-3’; CUDR/P2: 5’-taatgtaggtggcgatgagtt-3’.

Chromatin immunoprecipitation (CHIP) assay Cells were cross-linked with 1% (v/v) formaldehyde (Sigma) for 10 min at room temperature and stopped with 125 mM glycine for 5 min. Crossed-linked cells were washed with phosphate-buffered saline, resuspended in lysis buffer, and sonicated for 8-10 min in a SONICS VibraCell to generate DNA fragments with an average size of 500 bp or so.Chromatin extracts were diluted 5-fold with dilution buffer, pre-cleared with Protein-A/G-Sepharose beads, and immunoprecipitated with specific antibody on Protein-A/G-Sepharose beads. After washing, elution and de-cross-linking, the ChIP DNA was detected by either traditional PCR.

Chromosome conformation capture (3C) –chromatin immunoprecipitation (ChIP) assay（ChIP-3C/ChIP-Loop assays) Antibody-specific immunoprecipitated chromatin was obtained as described above for ChIP assays. Chromatin still bound to the antibody-Protein-A/G-Sepharose beads were resuspended in 500 µl of 1.2× restriction enzyme buffer at 37 °C for 1 h. 7.5 µl of 20% SDS was added, the mixture was incubated for 1 h, followed by addition of 50 µl of 20% Triton X-100, and then incubation for an additional 1 h. Samples were then incubated with 400 units of selected restriction enzyme at 37 °C overnight. After digestion, 40 µl of 20% SDS was added to the digested Chromatin, and the mixture was incubated at 65 °C for 10 min. 6.125 ml of 1.15× ligation buffer and 375 µl of 20% Triton X-100 was added, the mixture was incubated at 37 °C for 1 h, and then 2000 units of T4 DNA ligase was added at 16 °C for a 4-h incubation. Samples were then de-cross-linked at 65 °C overnight followed by phenol-chloroform extraction and ethanol precipitation. After purification, the ChIP-3C material was detected for long range interaction with specific primers. All primers had to be within a region of ±150 bp from the restriction enzyme digestion site. PCR products were amplified with AccuPrime Tag High Fidelity DNA Polymerase (Invitrogen) for 35 cycles. PCR products were run on a 2% agarose gel. Each validation experiment was repeated at least twice.

Dual Luciferase Reporter Assay Cells($1x10^5$/well of a six-well plate) were transiently transfected with 1 µg of luciferase construct and 0.1 µg of pRL-tk (promega) or indicated plasmids with the use of the Lipofectiamine™ 2000

(Invitrogen) . After incubation for 24 h, the cells were harvested with Passive Lysis Buffer (Promega), and luciferase activities of cell extracts were measured with the use of the Dual luciferase assay system (Promega) according to manufacturer's instructions. luciferase activity was measured and normalized for transfection efficiency with Renilla luciferase activity.Transfection was performed with at least three different batches of each reporter plasmid.

Nuclear Run on assay Nuclear run-on was performed by supplying biotin-probe to nuclei, and labeled transcripts were bound to streptavidin-coated streptavidin-agarose Resin.The cells are chilled, and the membranes are permeabilized or lysed. The nuclei are then incubated for a short time at 37 °C in the presence of nucleoside triphosphates (NTPs) and biotin labeled probe. The number of nascent transcripts on the gene at the time of chilling is thought to be proportional to the frequency of transcription initiation. To determine the relative number of nascent transcripts in each sample, the biotin labeled RNA is purified and hybridized to a membrane containing immobilized DNA from the gene of interest. The amount of biotin activity that hybridizes to the membrane is approximately proportional to the number of nascent transcripts.

MSI detection through Dot blot (Slot blot) Dot blots can only confirm the presence or absence of a biomolecule or biomolecules which can be detected by the DNA probes. Various Biotin labling MSI probes(Biotin-MSIs) added individually to the wells where a vacuum sucks the water (with NaOH and NH_4OAc) from underneath the membrane (nitrocellulose) as a dot and then is spotted through circular templates directly. The cells DNA is quantified and equal amounts are aliquoted into tubes. These are denatured (NaOH and 95° C) and can be hybridized with the membrane to allow for the detection of variation between samples. The signal can be detected by anti-Biotin Western blotting.

Cells proliferation CCK8 Assay Cells were synchronized in G0 phase by serum deprivation and then released from growth arrest by reexposure to serum, and then cells were grown in complete medium for assay according to the manufacturer

instruction. In brief, cells at a concentration 4×10^3 were seeded into 96-well culture plates in 100μl culture medium containing 10% heat-inactivated fetal calf serum(FCS).Before detected, add 10μg/well cell proliferation reagent CCK8 and incubate for 4 hours at 37°C and 5% CO_2 .Shake thoroughly for 1 min on shaker. Measure the absorbance of the samples against a background control as blank using a Microplate(ELISA) reader. Each sample was assayed in triplicates at daily intervals after seeding for up to 6 days consecutively. Cell growth curve was based on the corresponding the normalized values of OD450 and each point represents the mean of three independent samples.

Xenograft transplantation in vivo Four-weeks male athymic Balb/C mice were purchased from Shi laike company(Shanghi,China) and maintained in the Tongji animal facilities approved by the China Association for accreditation of laboratory animal care. The athymic Balb/C mouse per group were injected at the armpit area subcutaneously with suspension of 1×10^8 induced hepatocyte-like stem cells in 100μl of phosphate buffered saline. The mice were observed 8 weeks, and then sacrificed to recover the tumors. The wet weight of each tumor was determined for each mouse. A portion of each tumor was fixed in 4% paraformaldehyde and embedded in paraffin for histological hematoxylin-eosin(HE) staining. The use of mice for this work was reviewed and approved by the institutional animal care and use committee in accordance with China national institutes of health guidelines.

Supplementary FIGURE LEGENDS

FigureS1 The hepatoblast derived from human ES cells were subjected to transform onto liver cancer in CUDR overexpressed and CCL4 treated injury mouse liver. **A.** The representative analytic results of histological hematoxylin-eosin(HE) staining. A portion of each tumor was fixed in 4% paraformaldehyde and embedded in paraffin for HE staining. **B.** The representative analytic results of immunohistochemical staining. A portion of each tumor was fixed in 4% paraformaldehyde and embedded in paraffin for immunohistochemical staining with anti-AFP .

FigureS2 CUDR overexpression combined with inflammatory factor IL6 produced hepatocyte-like stem cells malignant transformation in *vitro* and in *vivo* **A**.The schematic illustrates a model of human stem cell line MEL-2 transfeced with CUDR overexpression or knockdown plasmids differentiation into hepatoblasts which were further differentiated into hepatocytes–like stem cells .Meanwile, the hepatoblasts were treated with IL6 till differentiation into hepatocyte-like cells, and the hepatocyte-like stem cells were further treated with IL6 for 10 days. **B.**S phase cells assay using BrdU. Each value was presented as mean±standard error of the mean (SEM),).**,P<0.01.**C.**Cells sphere formation ability.**D.** n *vivo* test in induced and treated hepatocytes–like stem cells. The representative analytic results of histological hematoxylin-eosin(HE) staining. A portion of each tumor was fixed in 4% paraformaldehyde and embedded in paraffin for histological hematoxylin-eosin(HE) staining. (original magnification×100).

Figure S3 CUDR enhances SUV39h2 expression in hepatocyte-like stem cells with IL6 treatment. **A.** METTL3 and FTO expression analysis by western blotting with anti-METTL3,anti-FTO. β-actin as internal control. **B.** Super-EMSA(gel-shift) with biotin-SUV39h2 cRNA probe and anti-N6A me antibody. The intensity of the band was examined by western blotting with anti-Biotin. **C**. Nuclear Run on followed by western blotting with anti-N6Ame antibody primer in IL6/12,TNFα

treated hepatocyte-like stem cells transfected with pCMV6-A-GFP,pCMV6-A-GFP-CUDR,pGFP-V-RS,pGFP-V-RS-CUDR. Histone 3 as internal control.

FigureS4 CUDR cooperates with IL6 to enhance NF-κB expression and phosphorylation. Chromosome conformation capture (3C)-chromatin immunoprecipitation (ChIP) with anti-H3K9me3,anti-RNA polII in IL6 untreated hepatocyte-like stem cells transfected with pCMV6-A-GFP, pCMV6-A-GFP-CUDR, pGFP-V-RS, pGFP-V-RS-CUDR.The PCR anlysis is applied for detecting NF-κB promoter-enhancer coupling product using NF-κB promoter and enhancer primers. The NF-κB promoter and enhancer as INPUT.

Figure S5 CUDR plus IL6 promotes miRs expression through pStat3 **A.** Chromatin Immunoprecipitation(CHIP**)** with anti-pStat3 followed by PCR with miR155,miR17,miR675,miR372,miR192 promoter primer in IL6 treated hepatocyte-like cells transfected with pCMV6-A-GFP,pCMV6-A-GFP-CUDR,pGFP-V-RS,pGFP-V-RS-CUDR. IgG CHIP as negative control. miR155,miR17,miR675,miR372,miR192 promoter DNA as INPUT. **B.** Nuclear run on analysis with Biotin probe of miR21,miR155,miR17,miR675,miR372,miR192 in IL6/12,TNFα treated hepatocyte-like cells transfected with pCMV6-A-GFP, pCMV6-A-GFP-CUDR,pGFP-V-RS,pGFP-V-RS-CUDR.β-actin as internal control.

Figure S6 CUDR plus IL6 alters long noncoding RNA expression through pStat3**.** Chromatin Immunoprecipitation(CHIP) with anti- pStat3 followed by PCR with MEG3, TERRA promoter primer in IL6 treated hepatocyte-like cells transfected with pCMV6-A-GFP,pCMV6-A-GFP-CUDR,pGFP-V-RS,,pGFP-V-RS-CUDR. IgG CHIP as negative control. CUDR,HOTAIR,MALAT1,MEG3,HULC,H19,TERRA promoter DNA as INPUT.

Supplementary FIGURE

FigureS1

A

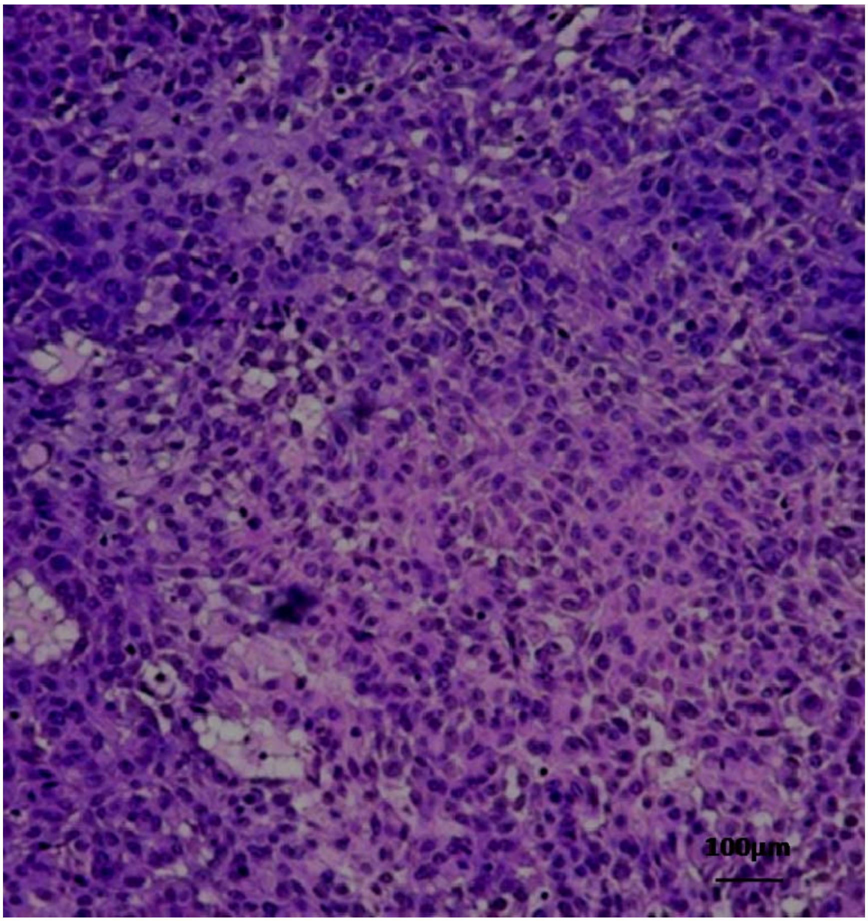

B

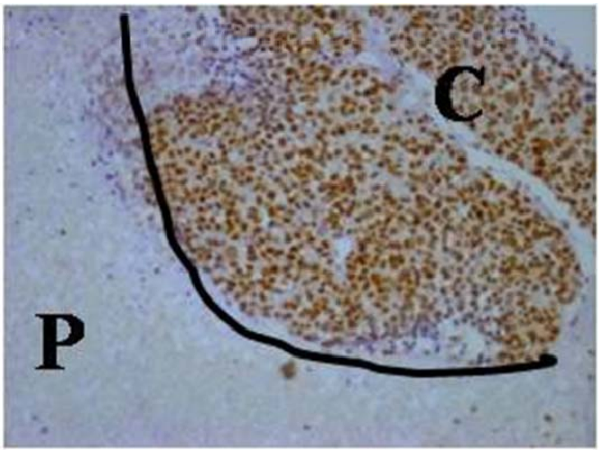

FigureS 2

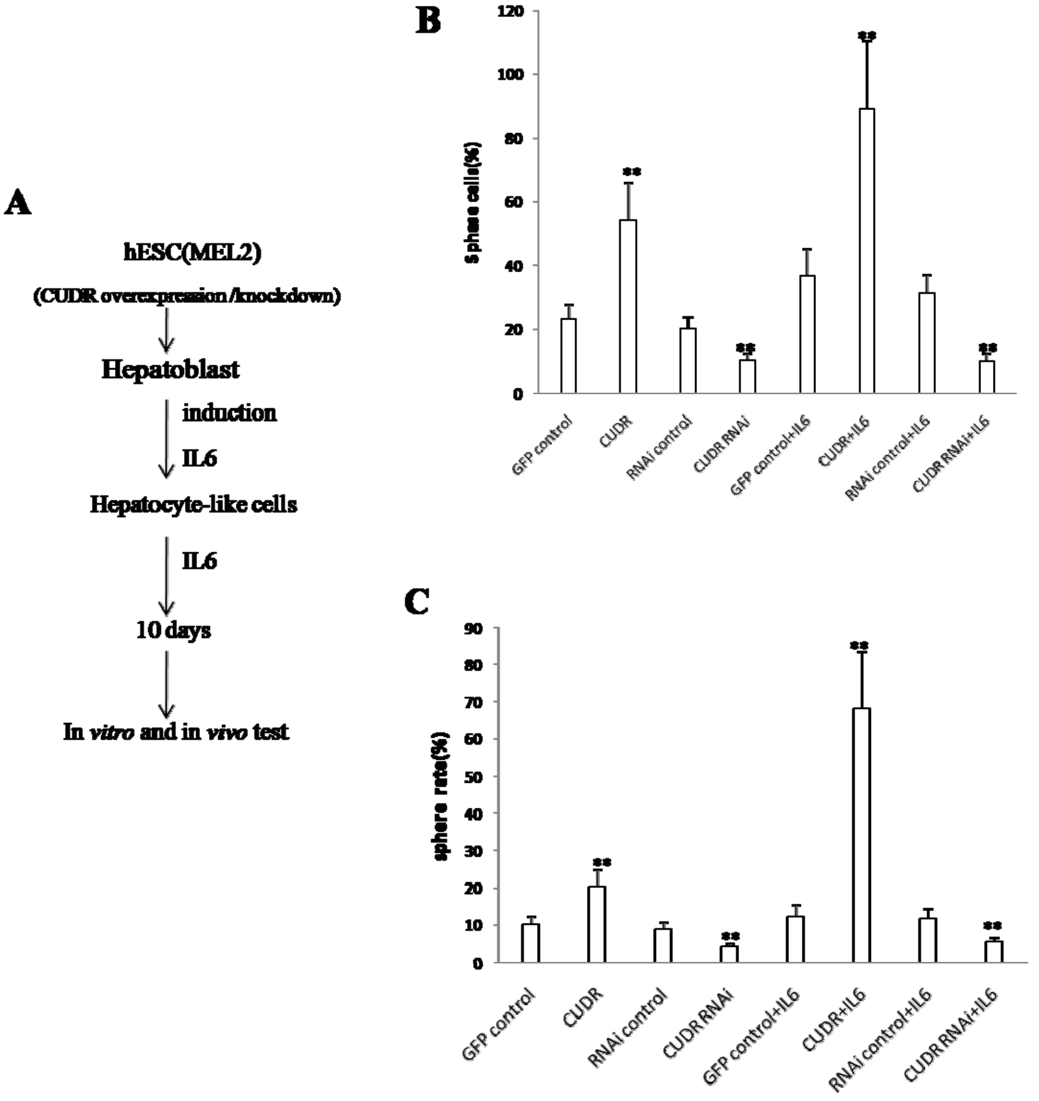
A
hESC(MEL2)
(CUDR overexpression /knockdown)
Hepatoblast
induction
IL6
Hepatocyte-like cells
IL6
10 days
In vitro and in vivo test
B
Sphere cells(%)
0
20
40
60
80
100
120
**
GFP control
CUDR
RNAi control
CUDR RNAi
GFP control+IL6
CUDR+IL6
RNAi control+IL6
CUDR RNAi+IL6
C
sphere rate(%)
0
10
20
30
40
50
60
70
80
90
**
GFP control
CUDR
RNAi control
CUDR RNAi
GFP control+IL6
CUDR+IL6
RNAi control+IL6
CUDR RNAi+IL6

D

Control

CUDR+IL6

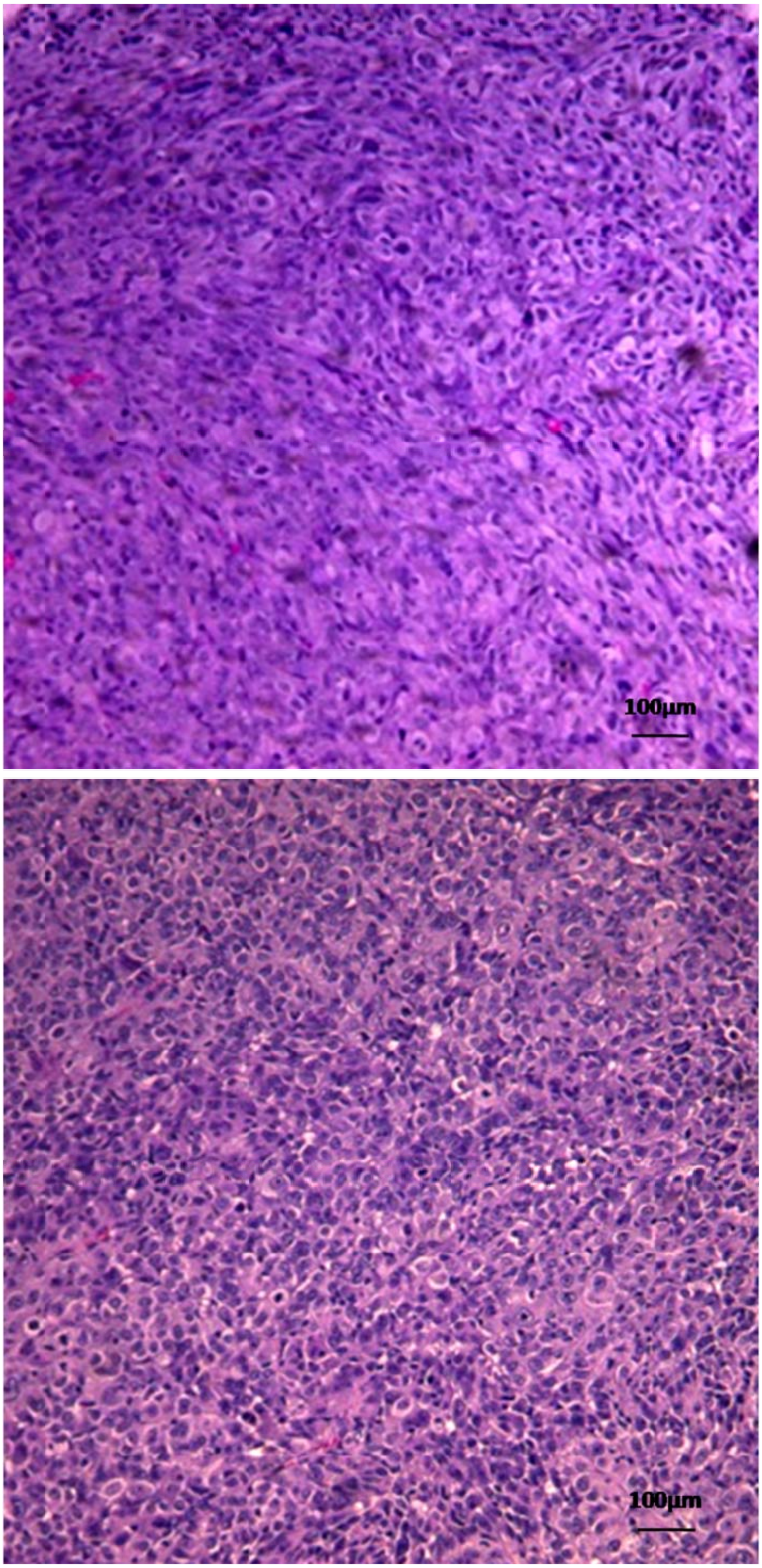

FigureS3

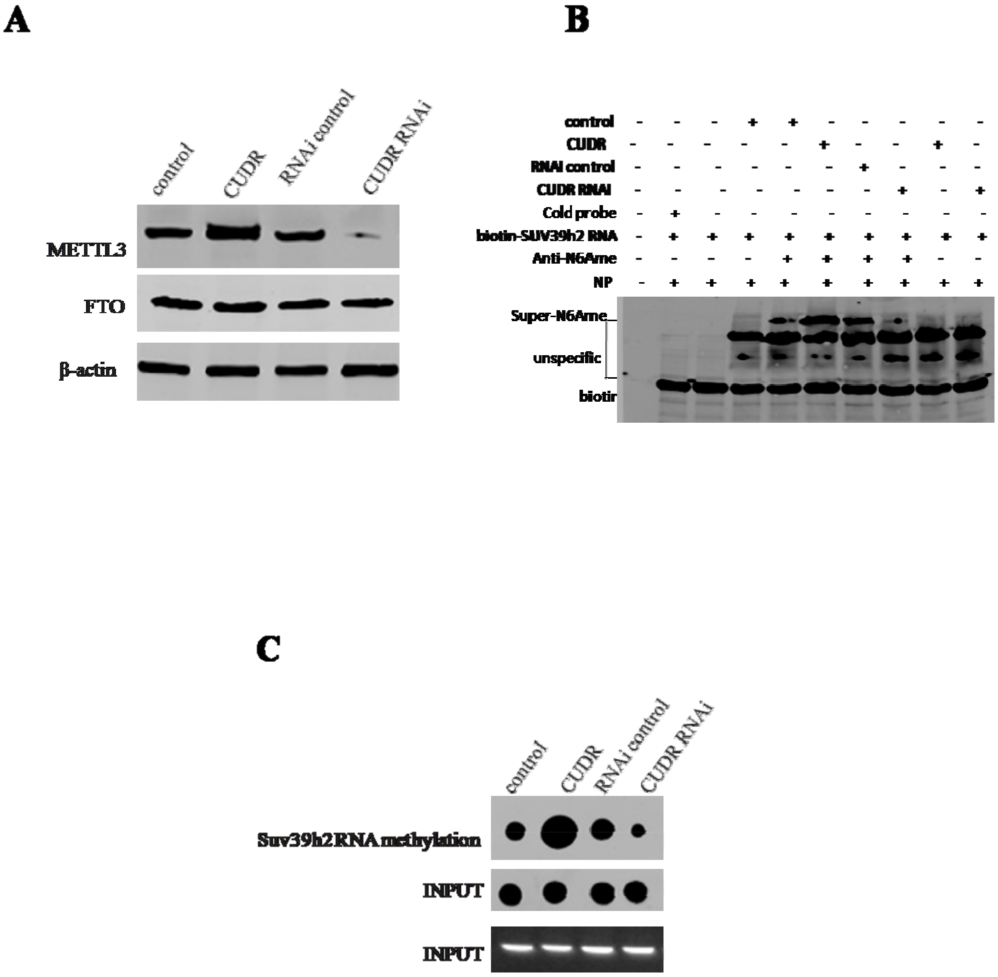
A
control
CUDR
RNAi control
CUDR RNAi
METTL3
FTO
β-actin
B
control
CUDR
RNAI control
CUDR RNAI
Cold probe
biotin-SUV39h2 RNA
Anti-N6Ame
NP
Super-N6Ame
unspecific
biotir
C
control
CUDR
RNAi control
CUDR RNAi
Suv39h2 RNA methylation
INPUT
INPUT

FigureS4

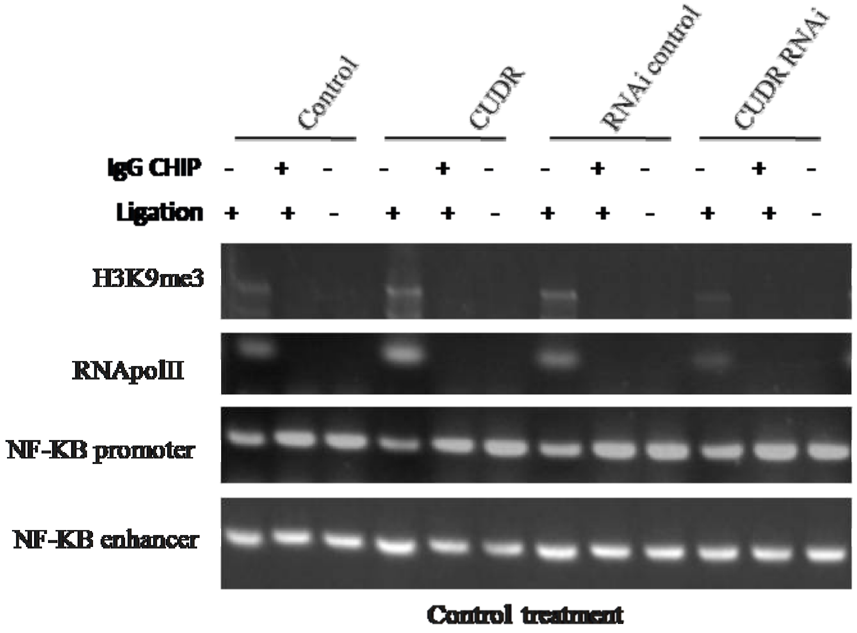
Control
CUDR
RNAi control
CUDR RNAi
IgG CHIP
Ligation
H3K9me3
RNApolII
NF-KB promoter
NF-KB enhancer
Control treatment

FigureS5

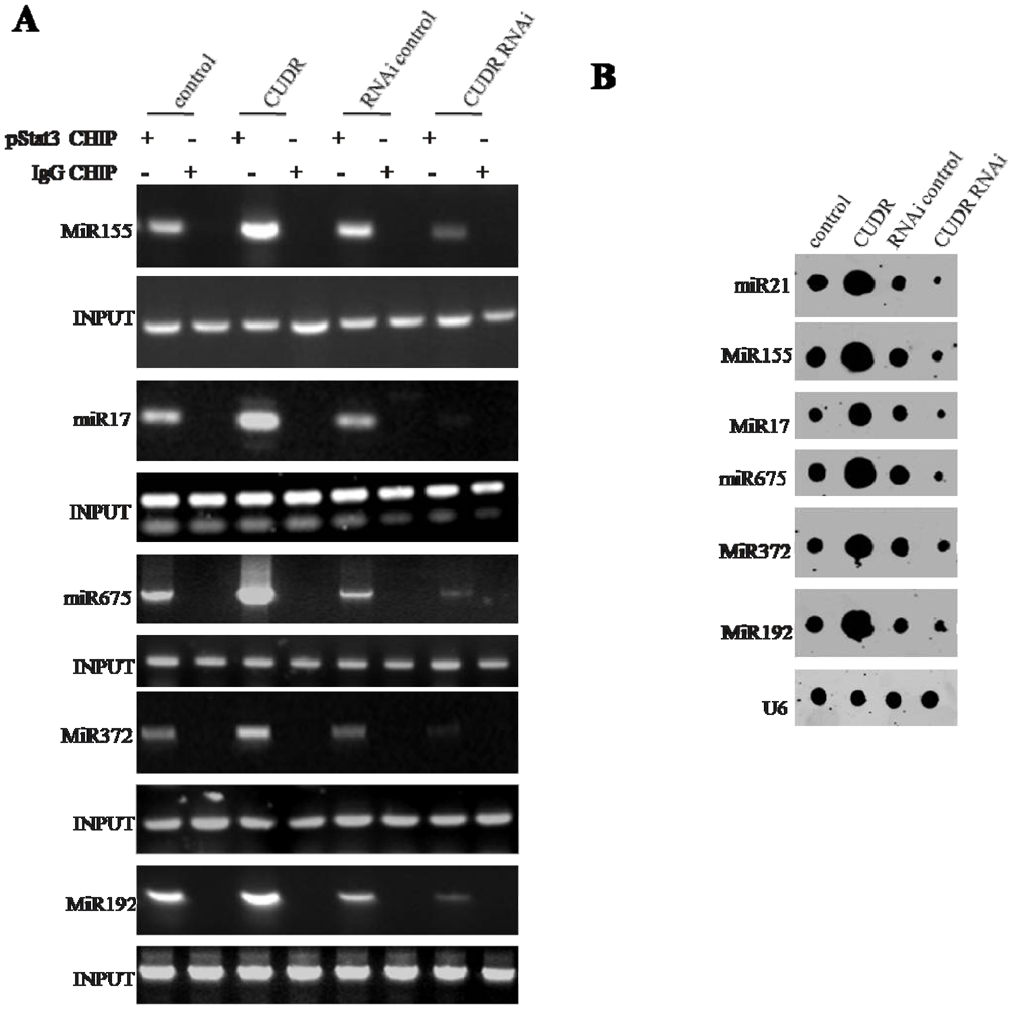
A
control
CUDR
RNAi control
CUDR RNAi
pStat3 CHIP
IgG CHIP
MiR155
INPUT
miR17
INPUT
miR675
INPUT
MiR372
INPUT
MiR192
INPUT
B
control
CUDR
RNAi control
CUDR RNAi
miR21
MiR155
MiR17
miR675
MiR372
MiR192
U6

FigureS6

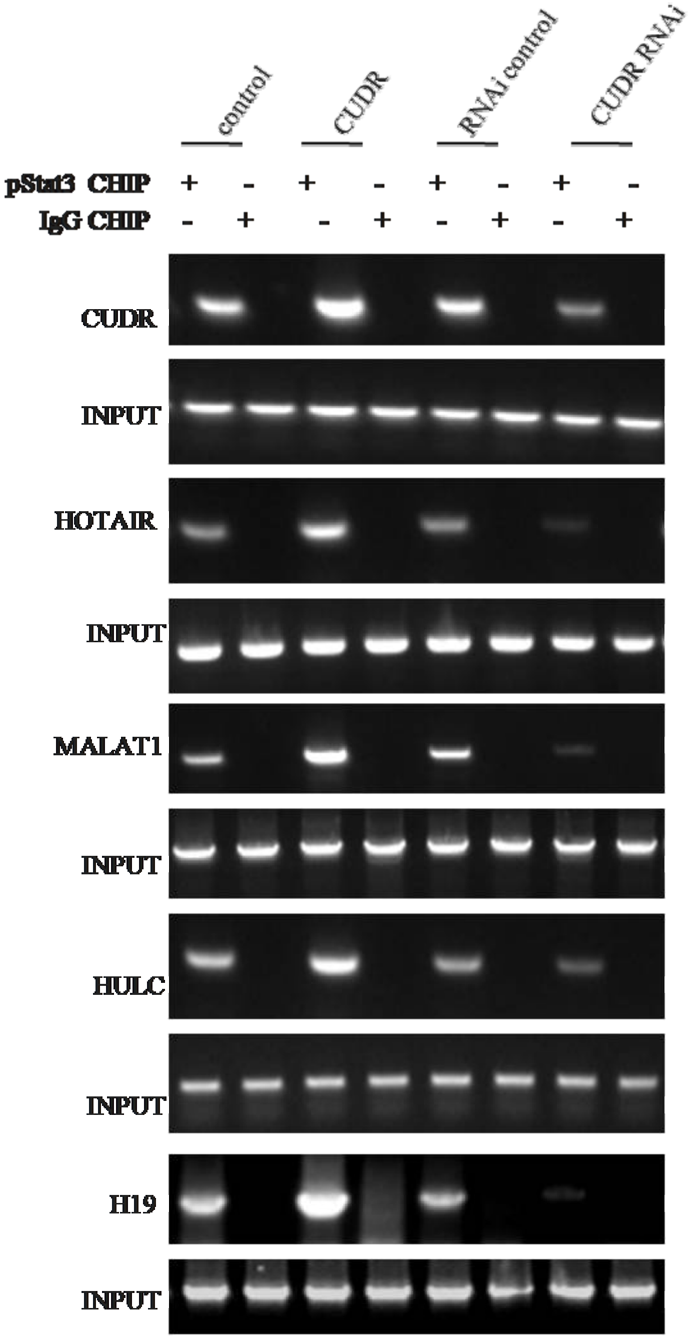
control
CUDR
RNAi control
CUDR RNAi
pStat3 CHIP + - + - + - + -
IgG CHIP - + - + - + - +
CUDR
INPUT
HOTAIR
INPUT
MALAT1
INPUT
HULC
INPUT
H19
INPUT
